# Metalloproteinase-Dependent and TMPRSS2-Independent Cell Surface Entry Pathway of SARS-CoV-2 Requires the Furin Cleavage Site and the S2 Domain of Spike Protein

**DOI:** 10.1128/mbio.00519-22

**Published:** 2022-06-16

**Authors:** Mizuki Yamamoto, Jin Gohda, Ayako Kobayashi, Keiko Tomita, Youko Hirayama, Naohiko Koshikawa, Motoharu Seiki, Kentaro Semba, Tetsu Akiyama, Yasushi Kawaguchi, Jun-ichiro Inoue

**Affiliations:** a Research Center for Asian Infectious Diseases, The Institute of Medical Science, The University of Tokyo, Tokyo, Japan; b Department of Life Science and Technology, Tokyo Institute of Technology, Yokohama, Japan; c Division of Cancer Cell Research, The Institute of Medical Science, The University of Tokyo, Tokyo, Japan; d Department of Life Science and Medical Bio-Science, Waseda University, Tokyo, Japan; e Laboratory of Molecular and Genetic Information, Institute for Quantitative Biosciences, The University of Tokyo, Tokyo, Japan; f Division of Molecular Virology, Department of Microbiology and Immunology, The Institute of Medical Science, The University of Tokyo, Tokyo, Japan; g Research Platform Office, The Institute of Medical Science, The University of Tokyo, Tokyo, Japan; Columbia University Medical College

**Keywords:** SARS-CoV-2, furin, membrane fusion, metalloproteinase, virus entry

## Abstract

The ongoing global vaccination program to prevent SARS-CoV-2 infection, the causative agent of COVID-19, has had significant success. However, recently, virus variants that can evade the immunity in a host achieved through vaccination have emerged. Consequently, new therapeutic agents that can efficiently prevent infection from these new variants, and hence COVID-19 spread, are urgently required. To achieve this, extensive characterization of virus-host cell interactions to identify effective therapeutic targets is warranted. Here, we report a cell surface entry pathway of SARS-CoV-2 that exists in a cell type-dependent manner and is TMPRSS2 independent but sensitive to various broad-spectrum metalloproteinase inhibitors such as marimastat and prinomastat. Experiments with selective metalloproteinase inhibitors and gene-specific small interfering RNAS (siRNAs) revealed that a disintegrin and metalloproteinase 10 (ADAM10) is partially involved in the metalloproteinase pathway. Consistent with our finding that the pathway is unique to SARS-CoV-2 among highly pathogenic human coronaviruses, both the furin cleavage motif in the S1/S2 boundary and the S2 domain of SARS-CoV-2 spike protein are essential for metalloproteinase-dependent entry. In contrast, the two elements of SARS-CoV-2 independently contributed to TMPRSS2-dependent S2 priming. The metalloproteinase pathway is involved in SARS-CoV-2-induced syncytium formation and cytopathicity, leading us to theorize that it is also involved in the rapid spread of SARS-CoV-2 and the pathogenesis of COVID-19. Thus, targeting the metalloproteinase pathway in addition to the TMPRSS2 and endosomal pathways could be an effective strategy by which to cure COVID-19 in the future.

## INTRODUCTION

Severe acute respiratory syndrome coronavirus 2 (SARS-CoV-2), the causative agent of coronavirus disease 2019 (COVID-19), was first recognized in late 2019 and led to the development of a global pandemic in 2020 (1). Two other human coronaviruses, SARS-CoV (2, 3) and Middle East respiratory syndrome coronavirus (MERS-CoV) (4), are also capable of inducing lethal pneumonia and systemic symptoms. However, SARS-COV-2 has been found to also exhibit enhanced pathogenicity and transmissibility (5, 6). Effective vaccines have been developed, and ongoing global vaccination programs have significantly curbed the spread of infection (7, 8). However, current vaccinations may provide imperfect protection, as new variants of the virus that can spread more easily and evade the host immunity achieved through vaccination have been reported (7, 9–11). Furthermore, although several drugs that may provide effective treatments for COVID-19 are currently approved (12, 13), it is unclear if daily life around the world will ever return to that of pre-COVID-19 times. Consequently, further extensive characterization of the virus and its interactions with host cells are required to develop vaccines and therapeutic agents that efficiently prevent infection from the new emerging variants.

The initiation of SARS-CoV-2 entry requires two steps after its spike (S) protein is cleaved into S1 and S2 by furin-like proteases expressed in virus-producing cells prior to viral release (14–16). First, the S protein binds to its receptor angiotensin converting enzyme 2 (ACE2) in the plasma membrane through its receptor-binding domain (RBD) (17, 18). Second, the S2 protein is cleaved to generate S2′ by either cell surface transmembrane serine protease 2 (TMPRSS2) or endosomal protease cathepsin-B/L (19, 20). This cleavage is called priming and exposes the fusion peptide within S2′, allowing it to stick into the plasma or endosomal membrane, resulting in fusion between the viral envelope and the cellular membrane (envelope fusion). This fusion allows viral RNA to enter the cytoplasm, where it replicates. Whether SARS-CoV-2 uses the plasma membrane, the endosomal pathway, or both is dependent on the cell type (20–22). Furin-mediated cleavage at the S1/S2 boundary leads to efficient viral entry into airway cells (15, 16), where the TMPRSS2-dependent surface entry route dominates endosomal entry (20, 23).

In this study, we identified a cell surface entry pathway of SARS-CoV-2 that is TMPRSS2 independent but sensitive to various metalloproteinase inhibitors. Interestingly, the metalloproteinase-dependent pathway requires both the furin cleavage motif and the S2 domain of SARS-CoV-2, which is unique to SARS-CoV-2. These results suggest that cooperation between furin and some metalloproteinases could be crucial for SARS-CoV-2 spread and disease development *in vivo*. Consequently, targeting the metalloproteinase pathway in addition to the TMPRSS2 and cathepsin-B/L pathways could be an effective strategy to cure COVID-19.

## RESULTS

### TMPRSS2-independent membrane fusion induced by the S protein of SARS-CoV-2 is blocked by metalloproteinase inhibitors.

**FIG 1:**
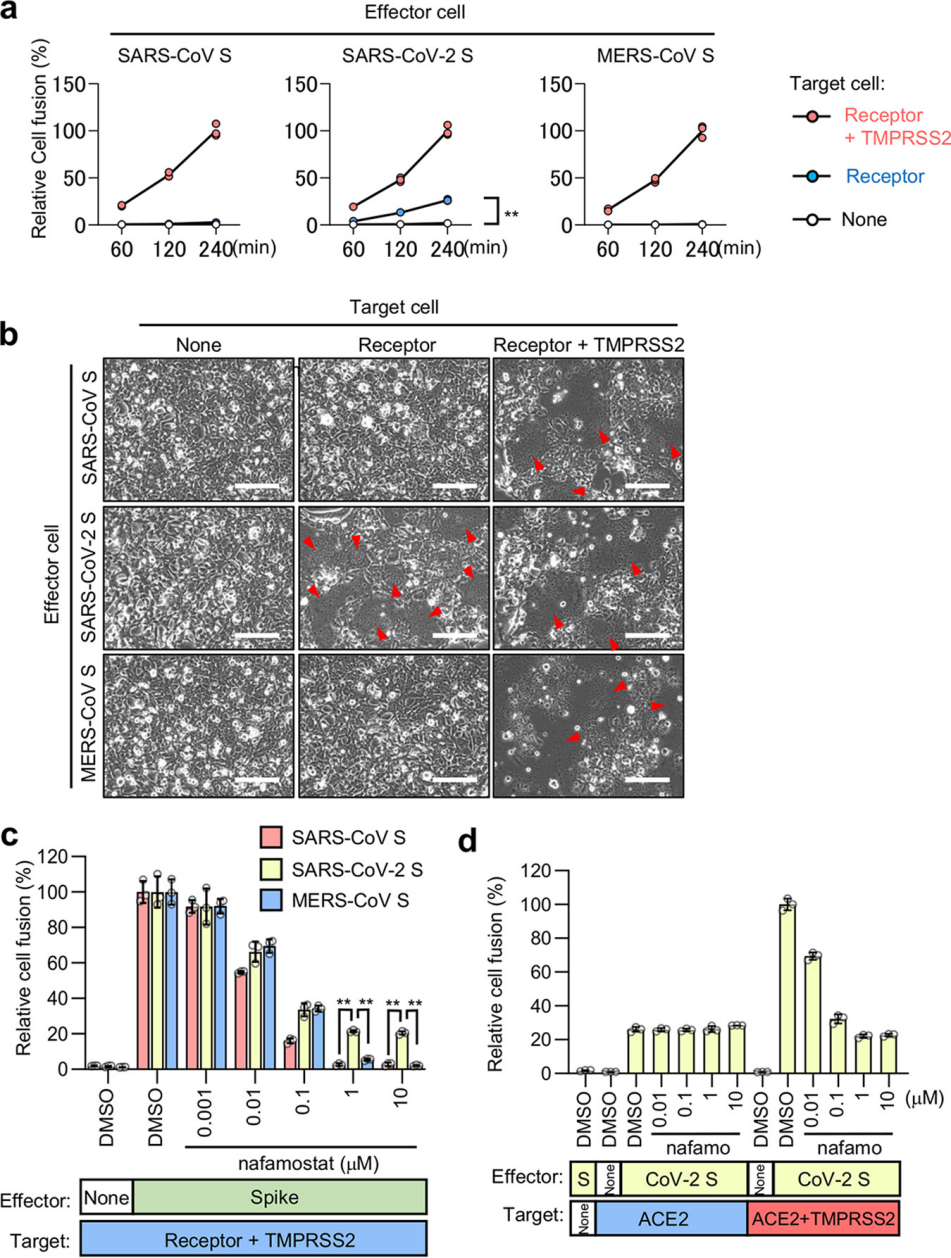
ACE2-dependent but TMPRSS2-independent membrane fusion activity of the SARS-CoV-2 S protein. (a) Cell fusion kinetics induced by the S proteins from SARS-CoV, SARS-CoV-2, and MERS-CoV were determined using the DSP assay. Target cells expressing ACE2 alone or together with TMPRSS2 were used for coculturing with effector cells expressing SARS-CoV S and SARS-CoV-2 S, and cells expressing CD26 alone or together with TMPRSS2 were used for coculturing with effector cells expressing MERS-CoV S. Relative cell fusion values were calculated by normalizing the RL activity of each coculture to that of the coculture with cells expressing both receptor and TMPRSS2 at 240 min, which was set to 100%. Values are means ± standard deviations (SD) (*n *=* *3/group). **, *P* < 0.01. (b) Phase-contrast images of S protein-mediated cell fusion 16 h after coculture. Red arrowheads indicate syncytium formation. Scale bars, 100 μm. (c) Effect of nafamostat on TMPRSS2-dependent cell fusion. Target cells expressing ACE2 with TMPRSS2 were used for coculturing with effector cells expressing SARS-CoV S and SARS-CoV-2 S, and cells expressing CD26 with TMPRSS2 were used for coculturing with effector cells expressing MERS-CoV S. Relative cell fusion values were calculated by normalizing the RL activity for each coculture to that of the coculture with cells expressing both receptor and TMPRSS2 in the presence of DMSO, which was set to 100%. Values are means ± SD (*n *=* *3/group). **, *P* < 0.01. (d) Effects of nafamostat on TMPRSS2-independent or -dependent cell fusion. Target cells expressing ACE2 alone or together with TMPRSS2 were used for coculturing with effector cells expressing SARS-CoV-2 S. Relative cell fusion values were calculated by normalizing the RL activity for each coculture to that of the coculture with cells expressing both ACE2 and TMPRSS2 in the presence of DMSO, which was set to 100%. Values are means ± SD (*n *=* *3/group). nafamo, nafamostat.

In this investigation, the screening system used to detect effective inhibitors of coronavirus infection included a dual split chimeric reporter protein (DSP)-mediated quantitative cell fusion assay between effector cells expressing S protein and target cells expressing either ACE2 (for SARS-CoV and SARS-CoV-2) (24) or CD26 (for MERS-CoV) (25), with or without TMPRSS2 (see Fig. S1a to d in the supplemental material). Endogenous TMPRSS2 expression was not detected in 293FT cells, while it was clearly detected in Caco-2 cells, in which TMPRSS2 is functionally active (20) (Fig. S1c). During analysis with the assay, a significant amount of ACE2-dependent but TMPRSS2-independent cell-cell fusion was induced by the S protein of SARS-CoV-2 but not by that of SARS-CoV or MERS-CoV (Fig. 1a and b). Consistent with this finding, the cell fusion with TMPRSS2 in the target cells induced by the S protein of SARS-CoV and MERS-CoV was completely blocked when TMPRSS2 was inhibited with nafamostat, while approximately 20% of the fusion by the SARS-CoV-2 S protein remained (Fig. 1c). This amount of residual fusion was almost equal to that induced by the SARS-CoV-2 S protein in the absence of TMPRSS2 (Fig. 1d).

10.1128/mbio.00519-22.1 FIG S1 Cell-based membrane fusion assay for coronavirus S proteins using the DSP reporter. (a) Method to monitor cell-cell membrane fusion mediated by the S protein of coronaviruses (24, 25). Effector cells (293FT cells expressing DSP8-11 and S protein) and target cells (293FT cells expressing DSP1-7 and receptor protein with TMPRSS2 for “cell fusion with TMPRSS2” [top] or receptor protein without TMPRSS2 for “cell fusion without TMPRSS2” [bottom]) were cocultured for 4 h. Both green fluorescent protein (GFP) (fluorescence) and *Renilla* luciferase (RL) (luminescence) signals were generated following DSP1-7 and DSP8-11 reassociation upon mixing of the cells during the assay. (b) Expression of S proteins in effector cells was detected using an anti-Flag tag antibody that binds to a Flag tag on the C terminus of S proteins (top). Tubulin was used as a control (bottom). S0, uncleaved S protein; S2, cleaved S2 domain of the S protein. (c) Expression of TMPRSS2 in Caco-2 and 293FT cells (top). Tubulin was used as a loading control (bottom). (d) Schematic diagram of split chimeric reporter proteins. DSP1-7 has the structure RL1–155-Ser-Gly-Gly-Gly-Gly-GFP1–156. DSP8-11 has the structure Met-GFP157–231-Gly-Gly-Gly-Gly-Ser-RL156–311. Since GFP1–156 contains the first seven β sheets and GFP157–231 contains the remaining four β sheets, the split proteins were called DSP1-7 and DSP8-11, respectively. DSP1-7 and DSP8-11 reassociate efficiently, resulting in the reconstitution of functional RL and GFP to generate luminescent and fluorescent signals, respectively. (e) Method to check whether compounds directly inhibit DSP activity without affecting cell-cell fusion (25). 293FT cells expressing DSP1-7 and DSP8-11 were treated with compounds for 4 h. RL activities of the preformed DSP1-7/DSP8-11 complex were measured to check whether the compounds directly inhibit RL activities without affecting cell-cell fusion. (f) Effect of metalloproteinase inhibitors on RL activity. Cells expressing DSP1-7 and DSP8-11 in the presence of inhibitors for 4 h were examined to determine whether compounds directly inhibit RL activities. (g) Effects of candidate compounds, including three tyrosine kinase inhibitors (sunitinib, PD-166285, and PD-173952), two checkpoint kinase inhibitors (PF-477736 and AZD-7762), a protein kinase C inhibitor (midostaurin), and a hormonal contraceptive (algestone), were analyzed. Effector cells expressing SARS-CoV-2 S were cocultured with target cells expressing ACE2 alone for the TMPRSS2-independent cell-cell fusion assay (blue) or cells expressing ACE2 with TMPRSS2 for the TMPRSS2-dependent cell-cell fusion assay (red) in the presence of candidate compounds for 4 h. Cells expressing DSP1-7 and DSP8-11 in the presence of candidate compounds for 4 h were examined to determine whether compounds directly inhibit RL activities (purple). Relative DSP activity was calculated by normalizing the RL activity for each condition to that of the control assay (DMSO alone; set to 100%) in panels e and f. Values are means ± SD (*n *=* *3/group). Download FIG S1, TIF file, 1.6 MB. Copyright © 2022 Yamamoto et al. 2022 Yamamoto et al. https://creativecommons.org/licenses/by/4.0/ This content is distributed under the terms of the Creative Commons Attribution 4.0 International license.

**FIG 2:**
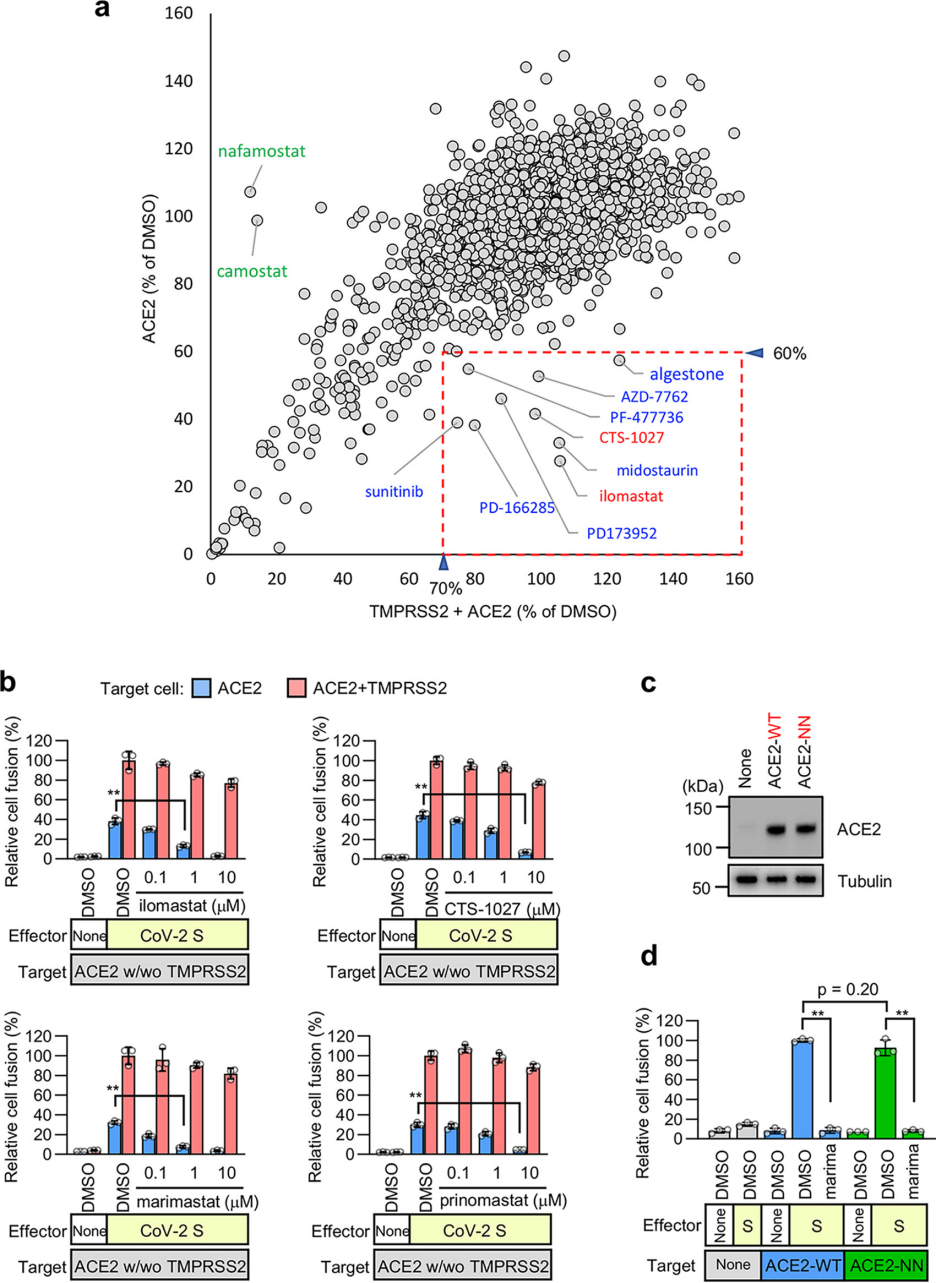
TMPRSS2-independent membrane fusion induced by the S protein of SARS-CoV-2 is blocked by various metalloproteinase inhibitors. (a) High-throughput screening of the Validated Compound Library (1,630 clinically approved compounds and 1,885 pharmacologically active compounds) obtained from the Drug Discovery Initiative (The University of Tokyo) by the DSP assay using the SARS-CoV-2 S protein. The *x* axis shows the relative cell fusion value using cells expressing both TMPRSS2 and ACE2 in the presence of each compound (1 μM in DMSO) (*n* = 1). The *y* axis shows the relative cell fusion value using cells expressing ACE2 alone in the presence of each compound (1 μM in DMSO) (*n* = 1). The relative cell fusion value was calculated by normalizing the RL activity for each compound to that of the control assay (DMSO alone; set to 100%). Each dot represents an individual compound. Dots in the red dashed box represent compounds that preferentially inhibit TMPRSS2-independent membrane fusion (<30% inhibition of the relative cell fusion value using the target cells expressing both TMPRSS2 and ACE2 and >40% inhibition of the relative cell fusion value using the target cells expressing ACE2 alone). The compound names for the candidates are indicated. (b) Effects of the metalloproteinase inhibitors on cell fusion in the cocultures of cells expressing SARS-CoV-2 S protein with those expressing ACE2 alone or in combination with TMPRSS2. Relative cell fusion values were calculated by normalizing the RL activity for each coculture to that of the coculture with cells expressing both ACE2 and TMPRSS2 in the presence of DMSO, which was set to 100%. Values are means ± SD (*n *=* *3/group). (c) Expression of ACE2 in target cells (top). Tubulin was used as a loading control (bottom). ACE2-WT, wild-type ACE2; ACE2-NN, enzymatically inactive ACE2 with H374N and H378N mutations. (d) Effects of the metalloproteinase inhibitor on cell fusion in the cocultures of cells expressing SARS-CoV-2 S protein with those expressing wild-type ACE2 (ACE2-WT) or enzymatically inactive ACE2 (ACE2-NN). Relative cell fusion values were calculated by normalizing the RL activity for each coculture to that of the coculture with cells expressing wild-type ACE2 in the presence of DMSO, which was set to 100%. Values are means ± SD (*n *=* *3/group). marima, 1 μM marimastat. **, *P* < 0.01.

To explore the mechanism of TMPRSS2-independent membrane fusion, we screened the Validated Compound Library (1,630 clinically approved compounds and 1,885 pharmacologically active compounds, https://www.ddi.u-tokyo.ac.jp/en/#5). To identify compounds that preferentially inhibited TMPRSS2-independent fusion, we chose for further validation compounds that limited the fusion without TMPRSS2 (*y* axis) by less than 60% and allowed the fusion with TMPRSS2 (*x* axis) by more than 70% (Fig. 2a). The metalloproteinase inhibitors ilomastat and CTS-1027 preferentially inhibited the TMPRSS2-independent fusion (Fig. 2b) without affecting the preformed luciferase complex, confirming the specificity for cell fusion (Fig. S1e and f). However, other compounds shown in the red dashed box in Fig. 2a inhibited both the TMPRSS2-dependent and -independent fusions to similar degrees (Fig. S1g). These data suggest that the metalloproteinase-dependent cell surface entry pathway (the metalloproteinase pathway) may be unique to SARS-CoV-2 among human pathogenic coronaviruses with high mortality rates. Considering that metalloproteinase inhibitors could thus possibly be used as prophylactic or therapeutic agents for COVID-19, we further demonstrated that marimastat (26) and prinomastat (27) (whose safety was previously confirmed in clinical trials, such as the clinical trial of CTS-1027 [https://clinicaltrials.gov/ct2/show/results/NCT01273064]) can preferentially block the TMPRSS2-independent fusion induced by the SARS-CoV-2 S protein (Fig. 2b; Fig. S1f). Interestingly, an enzymatically inactive mutant of ACE2 (ACE2-NN) (28) induced membrane fusion as did wild-type ACE2 (ACE2-WT), a member of the metalloproteinase family. Furthermore, marimastat completely inhibited both fusion reactions, suggesting that the TMPRSS2-independent membrane fusion depends on metalloproteinases other than ACE2 (Fig. 2c and d).

### The metalloproteinase pathway is SARS-CoV-2 specific and cell type dependent.

**FIG 3:**
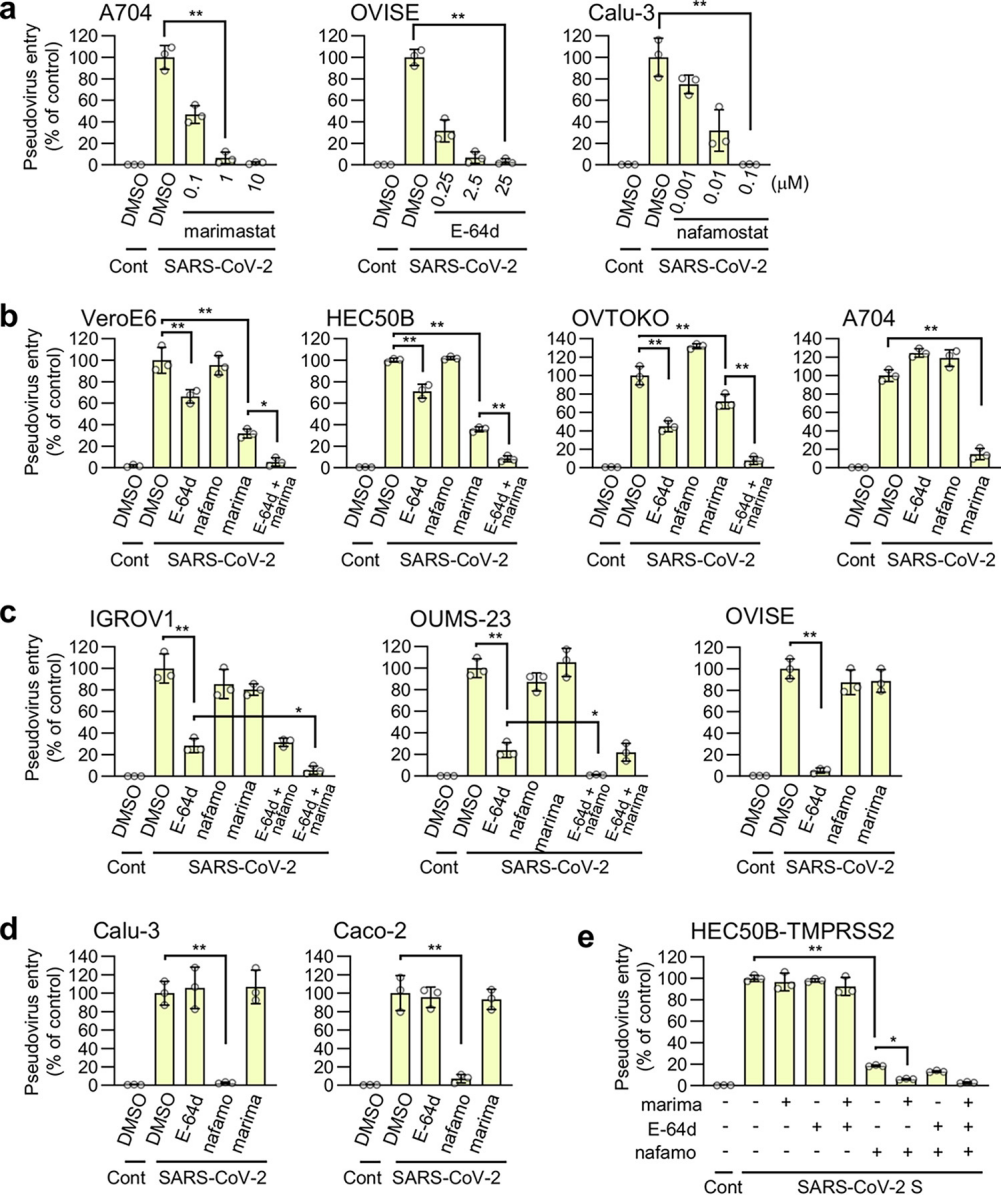
The metalloproteinase-dependent viral entry pathway is cell type dependent. The effects of drugs on the entry of SARS-CoV-2 S-bearing vesicular stomatitis virus (VSV) pseudotype virus produced by 293T cells are shown. The relative pseudovirus entry was calculated by normalizing the FL activity for each condition to the FL activity of cells infected with SARS-CoV-2 S-bearing pseudovirus in the presence of DMSO alone, which was set to 100%. Values are means ± SD (*n *=* *3/group). *, *P* < 0.05; **, *P* < 0.01. Cont, control (cells infected with pseudovirus without S protein); SARS-CoV-2, cells infected with SARS-CoV-2 S-bearing pseudovirus; E-64d, 25 μM E-64d; nafamo, 10 μM nafamostat; marima, 1 μM marimastat. (a) Effects of marimastat, E-64d, and nafamostat on pseudovirus entry in A704, OVISE, and Calu-3 cells, respectively. (b to e) Effects of a single drug treatment or a combination treatment on pseudovirus entry in VeroE6, HEC50B, OVTOKO, and A704 cells (b), IGROV1, OUMS-23, and OVISE cells (c), Calu-3 and Caco-2 cells (d), and HEC50B-TMPRSS2 cells (e).

We investigated whether the metalloproteinase pathway exists in SARS-CoV-2 S-bearing vesicular stomatitis virus (VSV) pseudovirus. The pseudovirus entry into the A704 cells (human kidney) was entirely blocked by 1 μM marimastat (Fig. 3a), indicating that the metalloproteinase pathway is involved in the entry of the virus and that 1 μM marimastat could be used to determine if the pathway exists in other cells. Similarly, all entry pathways in OVISE cells (human ovary) were blocked by 25 μM E-64d (endosomal cathepsin-B/L inhibitor), and the entry pathways in Calu-3 cells (human lung) were entirely blocked by 0.1 μM nafamostat (Fig. 3a), indicating that 25 μM E-64d or more than 0.1 μM nafamostat may be used to investigate the existence of the endosomal pathway or the TMPRSS2-dependent pathway, respectively, in other cells. Marimastat significantly inhibited pseudovirus entry into VeroE6 (African green monkey kidney), HEC50B (human endometrium), OVTOKO (human ovary), and A704 (Fig. 3b) cells. In addition to marimastat, virus entry was partially inhibited by E-64d, and the combination of marimastat and E-64d showed additive effects in VeroE6, HEC50B, and OVTOKO cells (Fig. 3b). These results suggest that the metalloproteinase and endosomal pathways are mutually independent. E-64d significantly inhibited the entry pathways of IGROV1 (human ovary) and OUMS-23 (human colon) cells and the overall entry pathway into OVISE cells (Fig. 3c). Interestingly, the E-64d-resistant entry (residual entry in the presence of E-64d) in IGROV1 cells was inhibited by the combination of marimastat and E-64d while the E-64d-resistant entry in OUMS-23 cells was inhibited by the combination of nafamostat and E-64d. These results indicate that the endosomal pathway dominates these cells while coexisting with either the metalloproteinase or TMPRSS2 pathway. Nafamostat inhibited the overall entry pathways into Calu-3 and Caco-2 (human colon) cells (Fig. 3d). Together, these findings show that the metalloproteinase pathway exists in a cell type-dependent manner and coexists with the endosomal pathway in some cell lines. As we could not find cell lines with both metalloproteinase- and TMPRSS2-dependent pathways, we generated HEC50B cells ectopically expressing TMPRSS2 (HEC50B-TMPRSS2). In the HEC50B-TMPRSS2 cells, approximately 80% of the entry pathways were TMPRSS2 dependent, while the rest were predominantly metalloproteinase dependent (Fig. 3e). This indicates that the metalloproteinase pathway independently coexists with the TMPRSS2-dependent pathway. These results also suggest that there could be cells *in vivo* that naturally have both surface entry pathways.

### The metalloproteinase pathway requires both the furin cleavage site and the S2 region of the SARS-CoV-2 S protein.

**FIG 4:**
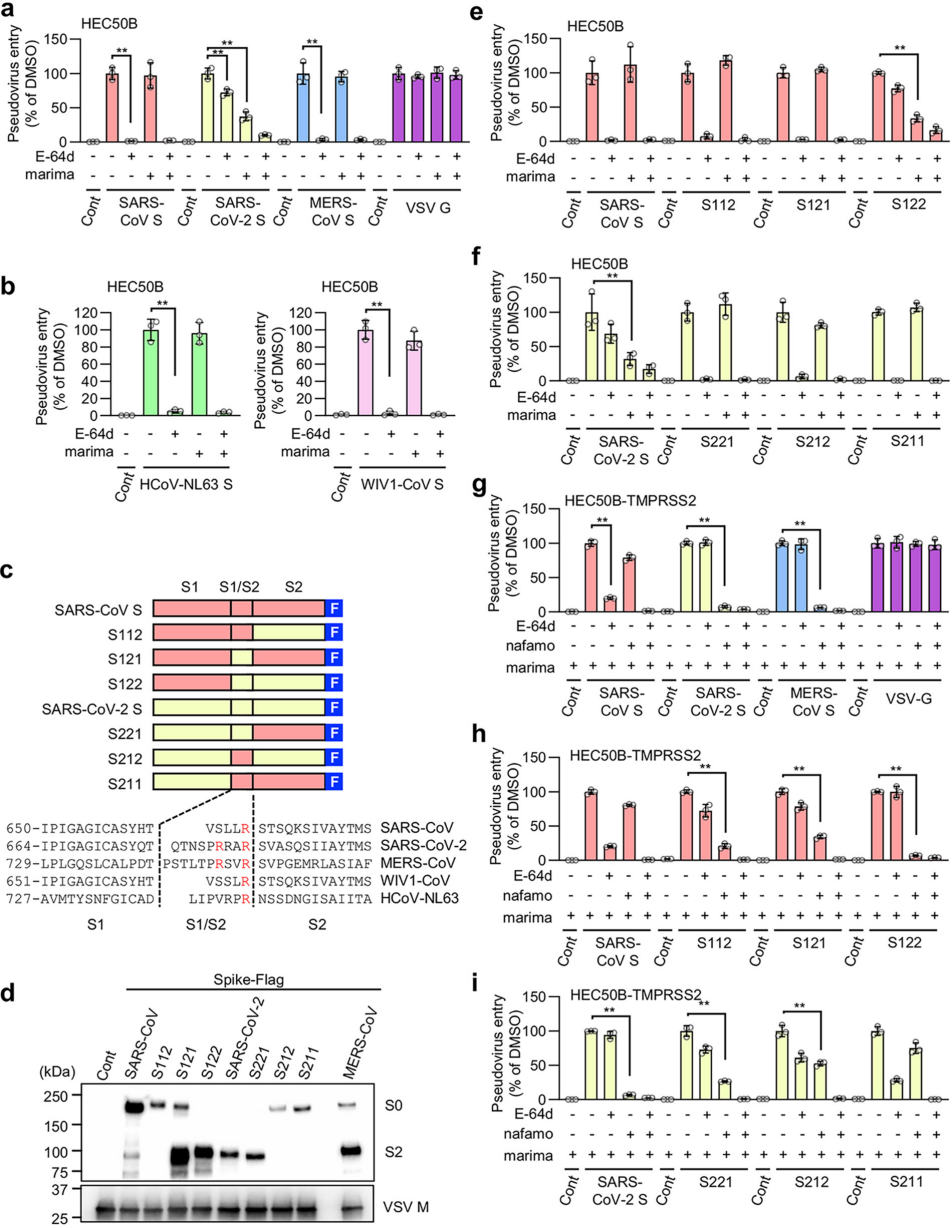
The metalloproteinase-dependent entry pathway requires both the furin cleavage site and S2 region of the SARS-CoV-2 S protein. The effects of drugs on the entry of S protein-bearing vesicular stomatitis virus (VSV) pseudotype virus produced by 293T cells are shown. The relative pseudovirus entry was calculated by normalizing the FL activity for each condition to the FL activity of cells infected with pseudovirus in the presence of DMSO alone, which was set to 100%. Values are means ± SD (*n *=* *3/group). **, *P* < 0.01. Cont, control (cells infected with pseudovirus without S protein); E-64d, 25 μM E-64d; marima, 1 μM marimastat; nafamo, 10 μM nafamostat. (a) Effects of E-64d and marimastat on the entry of pseudoviruses bearing SARS-CoV S, SARS-CoV-2 S, MERS-CoV S, or VSV G in HEC50B cells. (b) Effects of E-64d and marimastat on the entry of HCoV-NL63 S and WIV1-CoV S pseudovirus in HEC50B cells. (c) Schematic illustration of C-terminally Flag-tagged chimeric S proteins in which the S1, S1/S2 boundary, and S2 domain from SARS-CoV S (red) and SARS-CoV-2 S (yellow) are indicated (top). Amino acid sequences of the residues around the S1/S2 boundary of the coronaviruses (bottom). Numbers refer to the amino acid residues. F, Flag tag. Arginine residues in the S1/S2 cleavage site and furin cleavage motif are highlighted in red. (d) Expression of chimeric S protein in pseudoviruses. S proteins were detected using an anti-Flag tag antibody that binds to a Flag tag on the C terminus of the S proteins (top). Detection of the vesicular stomatitis virus matrix protein (VSV M) served as the control (bottom). Culture supernatants of 293T cells containing the pseudoviruses were centrifuged at 109,000 × *g* for 35 min at 4°C using a TLA100.3 rotor with an Optima TLX ultracentrifuge (Beckman Coulter, CA, USA), and the pellet was then lysed for Western blotting. S0, uncleaved S protein; S2, cleaved S2 domain of the S protein. (e and f) Effects of E-64d and marimastat on the entry of pseudoviruses bearing chimeric S proteins in HEC50B cells. (g) Effects of E-64d and nafamostat on the entry of pseudoviruses bearing SARS-CoV S, SARS-CoV-2 S, MERS-CoV S, or VSV G in HEC50B-TMPRSS2 cells in the presence of marimastat. (h and i) Effects of E-64d and nafamostat on the entry of pseudoviruses bearing chimeric S proteins in HEC50B-TMPRSS2 cells in the presence of marimastat.

Metalloproteinase-dependent cell-cell fusion was induced by the S protein of SARS-CoV-2 but not by that of SARS-CoV or MERS-CoV (Fig. 1a and b). In line with these results, metalloproteinase-dependent entry was observed only when the SARS-CoV-2 pseudovirus, but not SARS-CoV or MERS-CoV pseudovirus, was used in HEC50B (Fig. 4a), A704 (Fig. S2a), and VeroE6 cells (Fig. S2b). Furthermore, HCoV-NL63 and WIV1-CoV pseudoviruses, which like SARS-CoV-2 use ACE2 as their receptor, cannot utilize the metalloproteinase pathway (Fig. 4b). The ability to use the metalloproteinase pathway and sensitivities against various protease inhibitors are conserved among the variants of SARS-CoV-2 we tested (Fig. S3).

10.1128/mbio.00519-22.2 FIG S2 The metalloproteinase-dependent entry pathway strictly requires both the furin cleavage site and the S2 region of S protein of SARS-CoV-2. The effects of the drugs on the entry of S protein-bearing vesicular stomatitis virus (VSV) pseudotype virus are shown. The relative pseudovirus entry was calculated by normalizing the FL activity for each condition to the FL activity of the cells infected with pseudovirus in the presence of DMSO alone, which was set to 100%. Values are means ± SD (*n *=* *3/group). **, *P* < 0.01. Cont, control (cells infected with pseudovirus without S protein); E-64d, 25 μM E-64d; marima, 1 μM marimastat; nafamo, 10 μM nafamostat. (a and b) Effects of E-64d and marimastat on the entry of pseudoviruses bearing SARS-CoV S, SARS-CoV-2 S, MERS-CoV S, or VSV G in A704 (a) and VeroE6 (b) cells. (c) Schematic illustration of C-terminally Flag-tagged S proteins of WIV1-CoV and HCoV-NL63 and amino acid sequences of the residues around the S1/S2 boundary of the coronaviruses (bottom). Numbers refer to amino acid residues. F, Flag tag. Arginine residues in the S1/S2 cleavage site and furin cleavage motif are highlighted in red. (d) Expression of S protein in pseudovirus S proteins were detected using an anti-Flag tag antibody that binds to a Flag tag on the C terminus of S proteins (top). The detection of VSV M served as a control (bottom). S0, uncleaved S protein; S2, cleaved S2 domain of the S protein. (e and f) Effects of E-64d and marimastat on the entry of pseudoviruses bearing chimeric S proteins in VeroE6 cells. (g) Effects of E-64d and nafamostat on the entry of pseudoviruses bearing SARS-CoV S, SARS-CoV-2 S, MERS-CoV S, or VSV G in VeroE6-TMPRSS2 cells. (h and i) Effects of E-64d and nafamostat on the entry of pseudoviruses bearing chimeric S proteins in VeroE6-TMPRSS2 cells. To establish VeroE6 cells expressing TMPRSS2 (VeroE6-TMPRSS2), recombinant pseudotype lentivirus expressing TMPRSS2 was produced using 293T cells with a VSV G-expressing plasmid. Cells infected with pseudotype viruses were selected with 300 μg/mL hygromycin for at least 1 week. Download FIG S2, TIF file, 1.2 MB. Copyright © 2022 Yamamoto et al. 2022 Yamamoto et al. https://creativecommons.org/licenses/by/4.0/ This content is distributed under the terms of the Creative Commons Attribution 4.0 International license.

10.1128/mbio.00519-22.3 FIG S3 Patterns of entry pathways were conserved in various variants of SARS-CoV-2. (a) Expression of WT or mutant SARS-CoV-2 S proteins with mutations present in B.1.1.7, B.1.351, B.1.617.1, and B.1.617.2 variants in the pseudoviruses. S proteins were detected using an anti-Flag tag antibody that binds to a Flag tag on the C terminus of S proteins (top). Detection of vesicular stomatitis virus matrix protein (VSV M) served as a control (bottom). S0, uncleaved S protein; S2, cleaved S2 domain of the S protein. (b) Effects of E-64d and marimastat on the entry of pseudoviruses bearing SARS-CoV-2 S in VeroE6 cells. E-64d, 25 μM E-64d; marima, 1 μM marimastat. (c) Effects of nafamostat on the entry of pseudovirus bearing SARS-CoV-2 S in VeroE6-TMPRSS2 cells. (d) Effects of E-64d and marimastat on the entry of pseudoviruses bearing SARS-CoV-2 S in HEC50B cells. E-64d, 25 μM E-64d; marima, 1 μM marimastat. (e) Effects of marimastat on the entry of pseudoviruses bearing SARS-CoV-2 S in A704 cells. (f) Effects of nafamostat on the entry of pseudoviruses bearing SARS-CoV-2 S in Calu-3 cells. The relative pseudovirus entry was calculated by normalizing the FL activity for each condition to the FL activity of cells infected with pseudovirus in the presence of DMSO alone, which was set to 100%. Values are means ± SD (*n *=* *3/group in panels b to f). Data were compared with those obtained from cells infected with pseudoviruses bearing variant SARS-CoV-2 S in the presence of DMSO alone. *, *P* < 0.05; **, *P* < 0.01. Cont, control (cells infected with a pseudovirus without S protein in panels b to f). Download FIG S3, TIF file, 1.0 MB. Copyright © 2022 Yamamoto et al. 2022 Yamamoto et al. https://creativecommons.org/licenses/by/4.0/ This content is distributed under the terms of the Creative Commons Attribution 4.0 International license.

The S proteins of SARS-CoV-2 and MERS-CoV have furin cleavage sites (Arg-X-X-Arg) in their S1/S2 boundary area, and they were efficiently cleaved during the preparation of the pseudovirus (Fig. 4c and d). In contrast, the S proteins of SARS-CoV, HCoV-NL63, and WIV1-CoV, which do not use the metalloproteinase pathway, do not have furin cleavage sites and are not cleaved to any notable degree (Fig. 4c and d; Fig. S2c and d). Given that the MERS-CoV S protein does not use the metalloproteinase pathway (Fig. 4a), even though it was efficiently cleaved, we speculated that furin-catalyzed S protein cleavage is a prerequisite but not sufficient for using the metalloproteinase pathway. To test this hypothesis, we generated pseudoviruses bearing chimeric S proteins in which the S1, S1/S2 boundary, and S2 domains were derived from either SARS-CoV or SARS-CoV-2 (Fig. 4c and d). As expected, the S2 fragment of the C-terminally Flag-tagged S protein was mainly detected when pseudoviruses bearing S proteins with the furin cleavage site (S121, S122, SARS-CoV-2 S, and S221) were analyzed (Fig. 4d). In contrast, uncleaved S protein (S0) was mainly detected when S proteins without the furin cleavage site (SARS-CoV S, S112, S212, and S211) were used (Fig. 4d). When only the S1/S2 boundary, or the S2 domain was replaced with the corresponding domain of SARS-CoV-2 in the SARS-CoV S protein (S121, S112), the metalloproteinase pathway did not appear (Fig. 4e and Fig. S2e). However, when both domains were replaced with the corresponding domains of SARS-CoV-2 (S122), the metalloproteinase pathway appeared (Fig. 4e; Fig. S2e). Furthermore, when either the S1/S2 or S2 domain was replaced with the corresponding domain of SARS-CoV in the SARS-CoV-2 S protein (S212, S221), the metalloproteinase pathway disappeared (Fig. 4f; Fig. S2f). These results indicate that both the S1/S2 boundary and the S2 domain of SARS-CoV-2 are strictly required for the virus to utilize the metalloproteinase pathway when the endosomal pathway coexists.

To compare the structural requirements needed for the S protein to use the metalloproteinase pathway with those needed to use the TMPRSS2 pathway in cells with the endosomal pathway as an alternative, we used HEC50B-TMPRSS2 and VeroE6 cells ectopically expressing TMPRSS2 (VeroE6-TMPRSS2). While both of these cells exhibited the TMPRSS2 and endosomal pathways depending on the source of S protein (Fig. 4g; Fig. S2g), HEC50B-TMPRSS2 cells in addition maintained a significant amount of the metalloproteinase pathway (approximately 20% of the total entry pathway) (Fig. 3e). Therefore, inhibition of the metalloproteinase pathway by marimastat will promote protein structural requirements for the S protein to use the TMPRSS2 pathway in HEC50B-TMPRSS2 cells (Fig. 4g to i). In both cells, SARS-CoV-2 predominantly used the TMPRSS2 pathway, while SARS-CoV used the endosomal pathway (Fig. 4g; Fig. S2g). However, nafamostat inhibited S112 and S121 pseudovirus entry partially, but more efficiently, than it did SARS-CoV entry, while it inhibited S122 virus entry almost completely (Fig. 4h; Fig. S2h). Furthermore, in comparison with its inhibition of SARS-CoV-2, nafamostat only partially inhibited S221 or S212 virus entry, while it scarcely inhibited S211 virus entry (Fig. 4i; Fig. S2i). These results indicate that the furin cleavage site and the S2 domain of SARS-CoV-2 additively contribute to the ability of the virus to use the TMPRSS2 pathway. Together, these results show that although both the metalloproteinase and the TMPRSS2 pathways undergo priming of the S protein at the cell surface, the structural requirements of the S protein for efficient priming differ between the metalloproteinase and TMPRSS2 pathways.

### Possible involvement of ADAM10 in the metalloproteinase-dependent entry of SARS-CoV-2.

**FIG 5:**
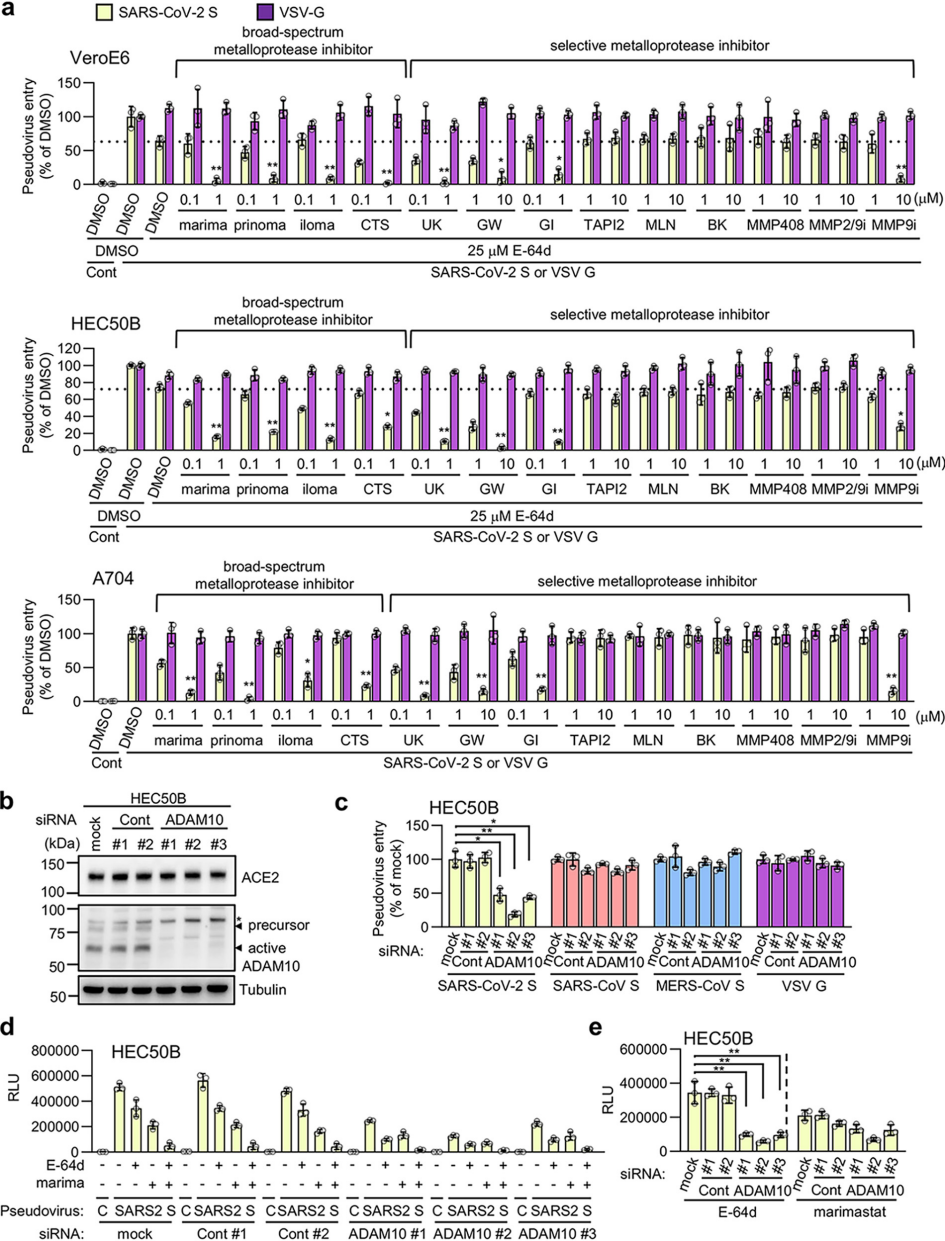
Possible involvement of ADAM-10 in the metalloproteinase-dependent entry of SARS-CoV-2. (a) Effects of metalloproteinase inhibitors on the entry of pseudoviruses bearing SARS-CoV-2 S or VSV G in VeroE6 and HEC50B cells in the presence of E-64d and in A704 cells in the absence of E-64d. The relative pseudovirus entry was calculated by normalizing the FL activity for each condition to the FL activity of cells infected with pseudovirus in the presence of DMSO alone, which was set to 100%. Values are means ± SD (*n *=* *3/group). Data were compared with those obtained from cells infected with pseudoviruses bearing SARS-CoV-2 S in the presence of E-64d for HEC50B and VeroE6 cells and in the presence of DMSO alone for A704 cells. *, *P* < 0.05; **, *P* < 0.01. Cont, control (cells infected with pseudovirus without S protein); marima, marimastat; prinoma, prinomastat; iloma, ilomastat; CTS, CTS-1027; UK, UK370106; GW, GW280264X; GI, GI254023X; MLN, MLN-4760; BK, BK-1361; MMP2/9i, MMP2/9 inhibitor I; MMP9i, MMP9 inhibitor I. (b) Effects of the ADAM10 knockdown on ACE2 (top), ADAM10 (middle), and tubulin (bottom) expression. HEC50B cells were transfected with two distinct control siRNAs or three distinct siRNAs against ADAM10 for 48 h. (c) Effect of the ADAM10 knockdown on the entry of pseudoviruses bearing SARS-CoV-2 S, SARS-CoV S, MERS-CoV S, or VSV G. HEC50B cells were transfected with siRNAs for 48 h and then infected with pseudoviruses. Relative pseudovirus entry was calculated by normalizing the FL activity for each condition to the FL activity of cells infected with pseudovirus in the absence of siRNA (mock), which was set to 100%. Values are means ± SD (*n *=* *3/group). *, *P* < 0.05; **, *P* < 0.01. (d and e) Effect of ADAM10 knockdown on the patterns of the entry pathways for SARS-CoV-2 S pseudovirus in HEC50B cells. HEC50B cells were transfected with siRNAs for 48 h and then infected with pseudoviruses in the presence of drugs. Values are means ± SD (*n *=* *3/group). **, *P* < 0.01. E-64d, 25 μM E-64d; marima, 1 μM marimastat. Data are displayed as the conditions of siRNA treatment (d) and drug treatment (e).

Several metalloproteinase inhibitors, including marimastat, prinomastat, ilomastat, and CTS-1027, which block SARS-CoV-2 S-mediated and TMPRSS2-independent cell-cell fusion (Fig. 2b), inhibited SARS-CoV-2 pseudovirus entry in VeroE6, HEC50B, and A704 cells (Fig. 5a). Since these inhibitors exhibited broad specificity (29–32), selective inhibitors were then used to narrow down the metalloproteinases involved in virus entry. Given that the VeroE6 and HEC50B cells had significantly E-64d-sensitive endosomal pathways (Fig. 3b), selective metalloproteinase inhibitors were tested in the presence of E-64d in these cells (Fig. 5a). Similar inhibitory patterns were observed in all three cell lines tested (Fig. 5a), and their viabilities were not affected (Fig. S4). These results suggest that the metalloproteinases involved in the pathway are likely to be common to all three cell lines. GW280264X (33) (ADAM10/17 inhibitor) and GI1254023X (33, 34) (MMP9/ADAM10 inhibitor) significantly inhibited the metalloproteinase pathway, whereas TAPI2 (35) (ADAM-17 inhibitor) and BK-1361 (36) (ADAM8 inhibitor) did not (Fig. 5a). This suggests that ADAM10 may be involved in the ADAM family. MMP408 (29) (MMP3/12/13 inhibitor) and MMP2/9 inhibitor I (37) scarcely affected virus entry, whereas UK370106 (38) (MMP3/12 inhibitor) and MMP9 inhibitor I (39) were significantly inhibitory (Fig. 5a), suggesting that MMP3/9/12/13 may not be crucial but that the unidentified metalloproteinase, which could be inhibited by UK370106 or MMP9 inhibitor I, may be involved. Consistent with the results of the cell fusion assay using enzymatically inactive ACE2 (Fig. 2c and d), MLN-4760 (40) (ACE2 inhibitor) did not inhibit virus entry (Fig. 5a), indicating that the catalytic activity of ACE2 is not involved. To further confirm the involvement of ADAM10, ADAM10 was depleted by small interfering RNA (siRNA) in HEC50B cells (Fig. 5b). An ADAM10 knockdown significantly inhibited SARS-CoV-2 pseudovirus entry, while the entry of SARS-CoV, MERS-CoV, and VSV G pseudoviruses was not affected (Fig. 5c), indicating that ADAM10 plays a role unique to SARS-CoV-2 in viral entry. Furthermore, we examined the effects of the ADAM10 knockdown on the entry pathway patterns by treating siRNA-transfected cells with either E-64d, marimastat, or a combination of the two. The combination treatment led to an additive effect for the single treatments, resulting in the complete inhibition of viral entry (Fig. 5d), indicating that the E-64d-resistant entry uses the metalloproteinase pathway while the marimastat-resistant entry uses the endosomal pathway. The ADAM10 knockdown significantly inhibited the metalloproteinase pathway (Fig. 5e, E-64d treatment, while the ADAM10 knockdown had only a modest effect on the endosomal pathway (Fig. 5e, marimastat treatment), indicating that ADAM10 is involved in the metalloproteinase-dependent entry pathway of SARS-CoV-2.

10.1128/mbio.00519-22.4 FIG S4 Effects of drugs on cell viabilities. (a to c) VeroE6 (a), HEC50B (b), and A704 (c) cells were treated with various drugs, and cell viability was analyzed using a CellTiter-Glo luminescent cell viability assay (G7570; Promega, WI, USA) 24 h after the treatment in accordance with the manufacturer's protocol. The relative cell viability was calculated by normalizing the FL activity for each condition to the FL activity of the cells in the presence of DMSO alone, which was set to 100%. Values are means ± SD (*n *=* *3/group). (d and e) HEC50B (d) and HEC50B-TMPRSS2 (e) cells were treated with various drugs for 3 days. Half of the culture supernatant was replaced daily with fresh medium containing the drugs. The relative cell viability was calculated by normalizing the FL activity for each condition to the FL activity of cells in the presence of DMSO alone, which was set to 100%. Values are means ± SD (*n *=* *6/group). Download FIG S4, TIF file, 0.9 MB. Copyright © 2022 Yamamoto et al. 2022 Yamamoto et al. https://creativecommons.org/licenses/by/4.0/ This content is distributed under the terms of the Creative Commons Attribution 4.0 International license.

Recently, it was reported that ACE2 shedding by ADAM17 promotes SARS-CoV-2 infection (41). While a CRISPR/Cas9-mediated ADAM17 knockout enhanced the accumulation of cellular ACE2 in HEC50B cells due to the inhibition of ACE2 shedding (Fig. S5a), SARS-CoV-2 pseudovirus entry was unexpectedly increased, and this was probably because of the enhanced binding of virus to the cell surface ACE2 (Fig. S5b). However, the patterns of the metalloproteinase and endosomal pathways were not changed by ADAM17 knockout (Fig. S5c), suggesting that ADAM17 may not be involved in metalloproteinase-dependent virus entry in HEC50B cells.

10.1128/mbio.00519-22.5 FIG S5 Patterns of the entry pathways of the pseudovirus bearing SARS-CoV-2 S were not affected by the ADAM17 knockout in HEC50B cells. (a) Effect of the ADAM17 knockout on ACE2 (top), ADAM17 (middle), and tubulin (bottom). (b) Effect of the ADAM17 knockout on the entry of the pseudoviruses bearing SARS-CoV-2 S. Values are means ± SD (*n *=* *3/group). **, *P* < 0.01. (c) Effect of the ADAM17 knockout on the patterns of the entry pathways of SARS-CoV-2 S pseudovirus in HEC50B cells. The relative pseudovirus entry was calculated by normalizing the FL activity for each condition to the FL activity of cells infected with pseudovirus in the presence of DMSO alone, which was set to 100%. Values are means ± SD (*n *=* *3/group). **, *P* < 0.01. E-64d, 25 μM E-64d; marima, 1 μM marimastat. To establish the ADAM17 knockout HEC50B cells, lentiviruses were produced by transfecting the lentiCRISPRv2 vector (catalog no. 52961; Addgene, MA, USA) with the following gRNA sequences: 5′-GCG AGG TAT TCG GCT CCG CG-3′ (Cont #1), 5′-GCT TTC ACG GAG GTT CGA CG-3′ (Cont #2), and 5′-ATG TTG CAG TTC GGC TCG AT-3′ (Cont #3) for the control experiments and 5′-AAC GTT CAG TAC TTG ATG TC-3′ (ADAM17 #1) and 5′-GGA CTT CTT CAC TGG ACA CG-3′ (ADAM17 #2), and 5′-CTT AAG GTG AGC CTG ACT CT-3′ (ADAM17 #3) for the establishment of ADAM17 knockout cells. Pooled HEC50B cells infected with pseudotype viruses were selected with 1 μg/mL puromycin for 1 week. Download FIG S5, TIF file, 0.6 MB. Copyright © 2022 Yamamoto et al. 2022 Yamamoto et al. https://creativecommons.org/licenses/by/4.0/ This content is distributed under the terms of the Creative Commons Attribution 4.0 International license.

### The metalloproteinase-dependent entry pathway of authentic SARS-CoV-2 is involved in syncytium formation and cytopathicity.

**FIG 6:**
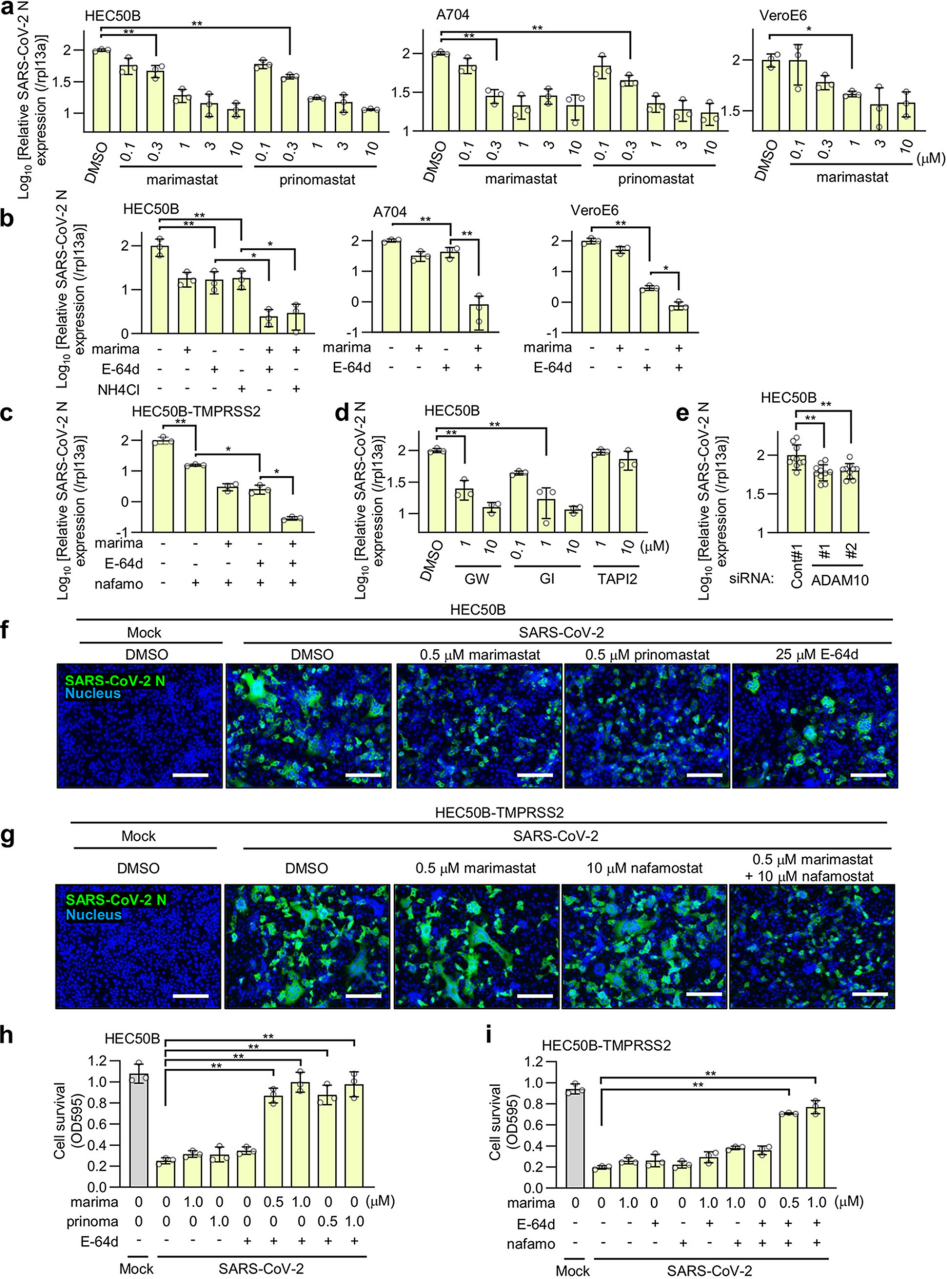
The metalloproteinase-dependent entry pathway of authentic SARS-CoV-2 is involved in syncytium formation and cytopathicity. The effects of the drugs on the cytoplasmic viral RNA after SARS-CoV-2 infection are shown. Cells were treated with inhibitors for 1 h and added with SARS-CoV-2 at an MOI of 0.01 for HEC50B and HEC50B-TMPRSS2 cells and at an MOI of 0.1 for VeroE6, Calu-3, and A704 cells. The relative amount of viral RNA in the cells was normalized to cellular *Rpl13a* mRNA expression. Values are means ± SD (*n *=* *3/group in panels a to d, *n *=* *10/group in panel e). *, *P* < 0.05; **, *P* < 0.01. (a) Effects of marimastat or prinomastat on SARS-CoV-2 infection in HEC50B, A704, and VeroE6 cells. (b) Effects of marimastat and the inhibitor of the endosomal pathway on SARS-CoV-2 infection in HEC50B, A704, and VeroE6 cells. marima, 1 μM marimastat; E-64d, 25 μM E-64d; NH4Cl, 10 mM NH_4_Cl. (c) Effect of marimastat, E-64d, and nafamostat on SARS-CoV-2 infection in HEC50B-TMPRSS2 cells. marima, 1 μM marimastat; E-64d, 25 μM E-64d; nafamo, 10 μM nafamostat. (d) Effects of selective metalloproteinase inhibitors on SARS-CoV-2 infection in HEC50B cells. GW, GW280264X; GI, GI254023X. (e) Effect of ADAM10 knockdown on SARS-CoV-2 infection in HEC50B cells. (f and g) Effects of drugs on SARS-CoV-2-induced syncytium formation in HEC50B (f) and HEC50B-TMPRSS2 (g) cells. Cells were stained with anti-SARS-CoV-2 N antibody (green) 24 h after infection. Nuclei were stained with Hoechst 33342 (blue). Scale bars, 200 μm. (h and i) Effects of drugs on SARS-CoV-2-induced cytopathicity in HEC50B (h) and HEC50B-TMPRSS2 (i) cells. marima, marimastat; prinoma, prinomastat; E-64d, 25 μM E-64d; nafamo, 10 μM nafamostat. Values are means ± SD (*n *=* *3/group). **, *P* < 0.01.

To confirm the involvement of the metalloproteinase pathway in authentic SARS-CoV-2 entry, we first evaluated the effects of marimastat and prinomastat on the level of cytoplasmic viral RNA after infection. Both inhibitors significantly suppressed SARS-CoV-2 infection (Fig. 6a). The 50% inhibitory concentration (IC_50_) values of marimastat and prinomastat were 160 nM and 130 nM in HEC50B cells and 150 nM and 250 nM in A704 cells, respectively. The IC_50_ value of marimastat in the VeroE6 cells was 340 nM. Nafamostat showed a marked inhibitory effect on Calu-3 cells but not on HEC50B, A704, or VeroE6 cells (Fig. S6a). In contrast, E-64d and NH_4_Cl, which inhibits endosome-lysosome system acidification (42), significantly suppressed SARS-CoV-2 infection in HEC50B, A704, and VeroE6 cells (Fig. S6b and c). This indicated that the endosomal pathway coexists with the metalloproteinase pathway to contribute to authentic SARS-CoV-2 infection in these cells. Combination treatments with E-64d/marimastat or NH_4_Cl/marimastat showed much stronger inhibitory effects than single treatments (Fig. 6b). Similarly, combination treatments with nafamostat/marimastat or nafamostat/E-64d showed stronger inhibitory effects than nafamostat treatment alone in HEC50B-TMPRSS2 cells (Fig. 6c). Furthermore, when all three drugs were combined, they had a much stronger inhibitory effect than the two-drug combinations (Fig. 6c). These results strongly suggest that drugs that block the metalloproteinase pathway are effective for COVID-19 treatment.

10.1128/mbio.00519-22.6 FIG S6 Effects of drugs on SARS-CoV-2 infection. (a) Effects of nafamostat on SARS-CoV-2 infection in Calu-3, HEC50B, A704, and VeroE6 cells. Values are means ± SD (*n *=* *3/group). **, *P* < 0.01. (b) Effects of E-64d on SARS-CoV-2 infection in HEC50B, A704, and VeroE6 cells. Values are means ± SD (*n *=* *3/group). *, *P* < 0.05; **, *P* < 0.01. (c) Effects of NH_4_Cl on SARS-CoV-2 infection in HEC50B cells. Values are means (*n *=* *2/group). The relative amount of viral RNA in the cells was normalized to cellular *Rpl13a* mRNA expression in panels a to c. (d) Phase-contrast images of syncytium formation 24 h after SARS-CoV-2 infection in the presence of inhibitors. Red arrowheads indicate syncytium formation. Scale bars, 100 μm. Download FIG S6, TIF file, 1.6 MB. Copyright © 2022 Yamamoto et al. 2022 Yamamoto et al. https://creativecommons.org/licenses/by/4.0/ This content is distributed under the terms of the Creative Commons Attribution 4.0 International license.

Next, we examined whether ADAM10 is involved in SARS-CoV-2 infection. GW280264X (ADAM10/17 inhibitor) and GI1254023X (MMP9/ADAM10 inhibitor) significantly suppressed SARS-CoV-2 infection, whereas TAPI2 (ADAM-17 inhibitor) did not (Fig. 6d). Moreover, the ADAM10 knockdown suppressed SARS-CoV-2 infection by approximately 40% (Fig. 6e), indicating that ADAM10 is partially involved.

The ability of SARS-CoV-2 to form syncytia and induce cytopathicity is thought to be related to its pathogenesis (43, 44). To determine whether the metalloproteinase-dependent pathway is involved in syncytium formation, we first used HEC50B cells as a representative for cells that predominantly use the metalloproteinase and endosomal pathways. Interestingly, the SARS-CoV-2-induced syncytium formation in HEC50B cells was significantly blocked by 500 nM marimastat and prinomastat but not notably affected by E-64d (Fig. 6f; Fig. S6d). These results indicate that the metalloproteinase-dependent pathway, but not the endosomal pathway, is crucial for syncytium formation, although both pathways similarly reduce viral infection (Fig. 6b). To investigate the involvement of the TMPRSS2 pathway in syncytium formation when it coexisted with the metalloproteinase pathway, we used HEC50B-TMPRSS2 cells. The syncytium formation in HEC50B-TMPRSS2 cells was not significantly inhibited by marimastat or nafamostat alone but was clearly inhibited by the combined treatment (Fig. 6g), suggesting that the metalloproteinase and TMPRSS2 cooperate to form syncytia. Next, we addressed the role of the metalloproteinase pathway in SARS-CoV-2-induced cytotoxicity. SARS-CoV-2-induced cytopathicity of HEC50B cells was not inhibited by E-64d, marimastat, or prinomastat alone but was significantly blocked by combination treatment with E-64d/marimastat or E-64d/prinomastat (Fig. 6h). In addition, SARS-CoV-2-induced cytopathicity of HEC50B-TMPRSS2 cells was not inhibited by E-64d, nafamostat, or marimastat alone but was significantly blocked when cells were treated with a combination of all three drugs (Fig. 6i). These results strongly suggest that the inhibition of the metalloproteinase pathway is crucial to block syncytium formation and cytopathicity *in vivo* and, consequently, that the metalloproteinase pathway is likely to be involved in the pathogenesis of COVID-19.

## DISCUSSION

In this study, we have demonstrated that SARS-CoV-2, unlike SARS-CoV or MERS-CoV, has a unique TMPRSS2-independent cell surface entry pathway that is sensitive to various metalloproteinase inhibitors. A significant proportion of the entry pathway was metalloproteinase dependent in A704 (kidney), HEC50B (endometrium), OVTOKO (ovary), and VeroE6 (kidney) cells. Only a small proportion was metalloproteinase-dependent in IGROV1 (ovary) cells, while the metalloproteinase pathway was not detected in OMUS-23 (colon), OVISE (ovary), Calu-3 (lung), and Caco-2 (colon) cells. These results indicate that the metalloproteinase pathway is cell type specific. Since SARS-CoV-2 can infect the kidney (45, 46) and induce acute kidney injury (47) in COVID-19 patients, the metalloproteinase pathway may contribute to the pathogenesis of COVID-19, especially multiple organ failure. The metalloproteinase pathway is thus a potential target for future COVID-19 therapies.

The S1/S2 boundary of SARS-CoV-2 contains the furin cleavage motif (Arg-X-X-Arg), while that of SARS-CoV contains only a single Arg. It has been reported that the motif greatly increases the efficiency of S1/S2 cleavage (15, 16), leading to enhanced viral transmission both *in vitro* (15, 16, 23) and *in vivo* (48, 49). This may be partially due to the enhanced availability of S2 to TMPRSS2, which could result from the dissociation of S1 (50, 51). We have shown that the furin cleavage motif is required for the metalloproteinase pathway, and we propose that the induction of metalloproteinase-mediated S2 priming is another role of furin-mediated S1/S2 cleavage in enhanced viral transmission. Interestingly, experiments using pseudoviruses bearing chimeric S proteins revealed that both the S1/S2 boundary and the S2 domain of SARS-CoV-2 S are essential for the metalloproteinase pathway. In contrast, the two domains of SARS-CoV-2 independently contributed to the TMPRSS2 pathway. This discrepancy may be partially due to the difference in the substrate recognition properties of the priming proteases in the two pathways. The pseudovirus bearing S112 (SARS-CoV S mutant, in which the S2 region was replaced with the corresponding domain of SARS-CoV-2) can use the TMPRSS2 pathway more efficiently than the SARS-CoV S pseudovirus, which suggests that TMPRSS2 may be partially accessible to the priming site in SARS-CoV-2 (C-terminally located from Arg815) but not to that in SARS-CoV (C-terminally located from Arg797) without S1 dissociation. In contrast, the putative priming protease in the metalloproteinase pathway, which may not be a metalloproteinase but a protease activated by metalloproteinases, can access the priming site only when the site occurs within the contextual characteristics of SARS-CoV-2 S2, and S1/S2 is cleaved to allow S1 dissociation. Determination of the priming site in the metalloproteinase pathway and identification of the critical amino acid residues generating the structural characteristics of SARS-CoV-2 S2 that allow metalloproteinase-dependent priming are required to understand its molecular mechanisms for the two distinct surface entry pathways. From an evolutionary perspective, SARS-CoV-2 acquired the metalloproteinase pathway by introducing mutations into the S2 region, which may have contributed to the SARS-CoV-2 pandemic. The function of point mutations in the S2 domain have not yet been fully analyzed. However, various point mutations in the S2 domain may play important roles in increasing the efficiency of infection and disease progression and the generation of highly infectious variants.

Using selective metalloproteinase inhibitors and ADAM10 knockdowns, we have demonstrated that ADAM10 plays an important role in the metalloproteinase pathway. ADAM10 is ubiquitously expressed in various tissues (https://www.proteinatlas.org/ENSG00000137845-ADAM10/tissue) and cell lines (https://www.proteinatlas.org/ENSG00000137845-ADAM10/celltype) and functionally regulates cell differentiation and proliferation by cleaving ligands and receptors (52). ADAM10 is thus likely to contribute to SARS-CoV-2 infection in various organs. ADAM17, similar to ADAM10, is also known as a metalloproteinase that can cleave common substrates such as Notch and HB-EGF (52). It has been reported that ACE2 shedding by ADAM17 promotes SARS-CoV-2 infection (41). Although we observed that ADAM17 depletion resulted in the cellular accumulation of ACE2, which is indicative of reduced ACE2 shedding, total SARS-CoV-2 pseudovirus infection was unexpectedly augmented and the relative contribution of the metalloproteinase pathway was not affected. ADAM10 and ADAM17 thus both play crucial but distinct roles in SARS-CoV-2 infection. It has recently been reported that ADAM9 inhibition decreases SARS-CoV-2 infection (53). Although the involvement of ADAM9 in viral entry is not clear, the results suggest that a group of metalloproteinases cooperate in the metalloproteinase pathway. This may indicate that the observed ADAM10 depletion-induced inhibition was a part of the maximum inhibition by various metalloproteinase inhibitors. A recent report also showed that MMP12 knockouts inhibited SARS-CoV-2 infection (54). However, MMP408 (29), an inhibitor of MMP12, did not prevent SARS-CoV-2 infection in various cell lines in this investigation, suggesting that metalloproteinases involved in the metalloproteinase pathway may differ in a cell type-dependent manner. Further studies are required to identify the functional metalloproteinases that are involved in the metalloproteinase pathway.

Various compounds are reported to inhibit SARS-CoV-2 entry *in vitro*. However, camostat (55), an inhibitor of TMPRSS2, and hydroxychloroquine (56, 57), an inhibitor of the endosomal pathway, have failed to show sufficient therapeutic efficacy in clinical trials. Our cytopathicity analysis revealed that cell death could not be inhibited unless all entry pathways were inhibited using inhibitor cotreatments for each pathway in HEC50B and HEC50B-TMPRSS2 cells. Therefore, future clinical trials on virus entry in which the TMPRSS2, metalloproteinase, and endosomal pathways are all efficiently blocked need to be conducted. We propose that to address this challenge, both marimastat and prinomastat should be utilized in clinical trials. The mean maximum plasma concentration (*C*_max_) at a reasonably well-tolerated dose was 590 nM for marimastat (26) and 680 nM for prinomastat (27). Furthermore, we demonstrated that these two drugs significantly inhibited SARS-CoV-2 infection at concentrations lower than their *C*_max_ values. These metalloproteinase inhibitors, in combination with other protease inhibitors, may effectively inhibit SARS-CoV-2 infection in various tissues and cure COVID-19. The results of this study may contribute to the development of COVID-19 treatments targeting viral entry pathways.

## MATERIALS AND METHODS

### Cell lines, viruses, and reagents.

All cell lines were cultured in accordance with the suppliers’ recommendations (see Table S1 in the supplemental material). To establish stable cell lines expressing the S proteins, ACE2, CD26, or TMPRSS2, recombinant pseudotype lentiviruses expressing one of the proteins were used as described previously (24). The SARS-CoV-2 isolate (UT-NCGM02/Human/2020/Tokyo) (58) was propagated in VeroE6-TMPRSS2 (JCRB1819) cells in Dulbecco’s modified Eagle medium (DMEM) containing 5% fetal bovine serum (FBS). siRNA (Table S2) was transfected using Lipofectamine RNAiMAX (Thermo Fisher Scientific, MA, USA) in accordance with the manufacturer's protocol. All protease inhibitors were dissolved in dimethyl sulfoxide (DMSO) at a concentration of 10 mM. See Table S3 for inhibitors.

10.1128/mbio.00519-22.7 TABLE S1 Cell lines used in this study. Download Table S1, DOCX file, 0.03 MB. Copyright © 2022 Yamamoto et al. 2022 Yamamoto et al. https://creativecommons.org/licenses/by/4.0/ This content is distributed under the terms of the Creative Commons Attribution 4.0 International license.

10.1128/mbio.00519-22.8 TABLE S2 siRNAs and primers used in this study. Download Table S2, DOCX file, 0.02 MB. Copyright © 2022 Yamamoto et al. 2022 Yamamoto et al. https://creativecommons.org/licenses/by/4.0/ This content is distributed under the terms of the Creative Commons Attribution 4.0 International license.

10.1128/mbio.00519-22.9 TABLE S3 Inhibitors used in this study. Download Table S3, DOCX file, 0.03 MB. Copyright © 2022 Yamamoto et al. 2022 Yamamoto et al. https://creativecommons.org/licenses/by/4.0/ This content is distributed under the terms of the Creative Commons Attribution 4.0 International license.

### Expression vector construction.

To construct expression vectors for ACE2, CD26, TMPRSS2, or S, the coding regions were cloned into a lentiviral transfer plasmid (CD500B-1; System Biosciences, CA, USA). Mutant ACE2 (ACE2-H374N/H378N) was constructed by PCR. Synthetic DNA corresponding to the codon-optimized S gene of SARS-CoV-2 (Wuhan-Hu-1; GenBank accession no. NC_045512.2), SARS-CoV-2 variants (B.1.1.7, EPI_ISL_601443; B.1.351, MZ747297.1; B.1.617.1, EPI_ISL_1704611; B.1.617.2, EPI_ISL_3189054), SARS-CoV (NC_004718.3), WIV1-CoV (KF367457.1), HCoV-NL63 (NC_005831.2), the chimeric S, and the Flag-tagged 5′-GGA GGC GAT TAC AAG GAT GAC GAT GAC AAG TAA-3′ (underlining indicates the Flag tag) at the 3′ end were all generated by Integrated DNA Technologies (IA, USA). Previously described synthetic DNA corresponding to the codon-optimized MERS-CoV S (NC_019843.3) (25) with a Flag tag at the 3′ end was used in this study.

### DSP assay to monitor membrane fusion.

The DSP assay was performed as described previously (24). Briefly, effector cells expressing S protein and target cells expressing CD26 or ACE2 alone or together with TMPRSS2 were seeded in 10-cm plates and incubated overnight (Fig. S1a and b). Cells were treated with 6 μM EnduRen (Promega), a substrate for *Renilla* luciferase (RL), for 2 h. To test the effect of inhibitor, 0.25 μL of compound library or 1 μL of selected inhibitor dissolved in DMSO was added to the 384-well plates (Greiner Bioscience, Frickenhausen, Germany). Next, 50 μL of each single-cell suspension (effector and target cells) was added to the 384-well plates by using a Multidrop dispenser (Thermo Fisher Scientific). After incubation at 37°C in 5% CO_2_ for 4 h, the RL activity was measured using a Centro xS960 luminometer (Berthold, Bad Wildbad, Germany).

### Western blotting.

Western blot analysis was performed as described previously (59). See Table S4 for antibodies.

10.1128/mbio.00519-22.10 TABLE S4 Antibodies used in this study. Download Table S4, DOCX file, 0.03 MB. Copyright © 2022 Yamamoto et al. 2022 Yamamoto et al. https://creativecommons.org/licenses/by/4.0/ This content is distributed under the terms of the Creative Commons Attribution 4.0 International license.

### Preparation of pseudotype VSV viral particles and infection experiments.

For producing a replication-deficient VSV, BHK cells expressing T7 RNA polymerase were transfected with T7 promoter-driven expression plasmids for VSV proteins (pBS-N/pBS-P/pBS-L/pBS-G) and pΔG-Luci (a plasmid encoding VSV genomic RNA which lacks the G gene and encodes firefly luciferase) as described previously (60, 61). At 48 h posttransfection, the supernatants were harvested. 293T cells were then transfected with an expression plasmid for S or VSV G by using calcium phosphate precipitation. At 16 h posttransfection, the cells were inoculated with a replication-deficient VSV at a multiplicity of infection (MOI) of 1. At 2 h postinfection, the cells were washed and further incubated for 16 h before the supernatant containing the pseudovirus was harvested. For the infection assay, cells were seeded in 96-well plates (2 × 10^4^ cells/well) and incubated overnight. The cells were pretreated with inhibitors for 1 h before the pseudovirus infection. Luciferase activity was measured at 16 h postinfection by using the Bright-Glo luciferase assay system or ONE-Glo luciferase assay system (Promega) and a Centro xS960 luminometer (Berthold).

### Quantification of intracellular SARS-CoV-2 RNA.

Cells were seeded in 96-well plates (5 × 10^4^ cells/well) and incubated overnight. Cells were treated with inhibitors for 1 h and added with SARS-CoV-2 at the MOIs indicated in the figure legends. Cell lysis and cDNA synthesis were performed at 24 h postinfection using a SuperPrep II cell lysis and RT kit for quantitative PCR (qPCR) (SCQ-401; Toyobo, Osaka, Japan) in accordance with the manufacturer’s instructions. Quantitative real-time reverse transcription (RT)-PCR for SARS-CoV-2 N and ribosomal protein L13a (*Rpl13a*) was performed using Thunderbird SYBR qPCR mix (Toyobo) with a CFX Connect real-time PCR detection system (Bio-Rad, CA, USA) at 95°C for 3 min, followed by 50 cycles of 95°C for 10 s and 60°C for 1 min. The level of *Rpl13a* mRNA expression in each sample was used to standardize the data. See Table S2 for primers.

### Immunofluorescence staining.

Cells were seeded in 24-well plates (1.5 × 10^5^ cells/well) and incubated overnight. Cells were treated with inhibitors for 1 h and then with SARS-CoV-2 at an MOI of 1. Cells were fixed at 24 h postinfection with 4% paraformaldehyde and permeabilized with 0.1% Triton X-100. Cells were incubated with primary antibody for 16 h at 4°C and detected with secondary antibody. Cell nuclei were stained with 1 μg/mL Hoechst 33342 (catalog no. 080-09981; Fujifilm Wako Pure Chemical). Fluorescent signals were detected using a BZ-X810 fluorescence microscope (Keyence, Osaka, Japan). See Table S4 for antibodies.

### Cytopathicity assay.

Cells were seeded in 24-well plates (1.5 × 10^5^ cells/well) and incubated overnight. Cells were treated with inhibitors for 1 h and then with SARS-CoV-2 at an MOI of 1. To maintain the drug concentration, half of the culture supernatant was replaced daily with fresh medium with drugs. Cells were fixed at 3 days postinfection with 4% paraformaldehyde and stained with 0.2% crystal violet. After washing four times with water, the wells were air dried, and crystal violet was dissolved with ethanol. The absorbance was measured at 595 nm using an iMark microplate reader (Bio-Rad).

### Statistical analysis.

Statistically significant differences between the mean values were determined using a two-tailed Student's *t* test. Dunnett's test and Tukey’s test were used for multiple comparisons. All data represent three independent experiments, and values represent the mean ± standard deviation, with a *P* of <0.05 considered statistically significant.
