# Supplementary figures and images for: Metalloproteinase-Dependent and TMPRSS2-Independent Cell Surface Entry Pathway of SARS-CoV-2 Requires the Furin Cleavage Site and the S2 Domain of Spike Protein
Source: mBio. 2022 Jun 16;13(4):e00519-22. doi: 10.1128/mbio.00519-22 (PMC9426510; doi:10.1128/mbio.00519-22)

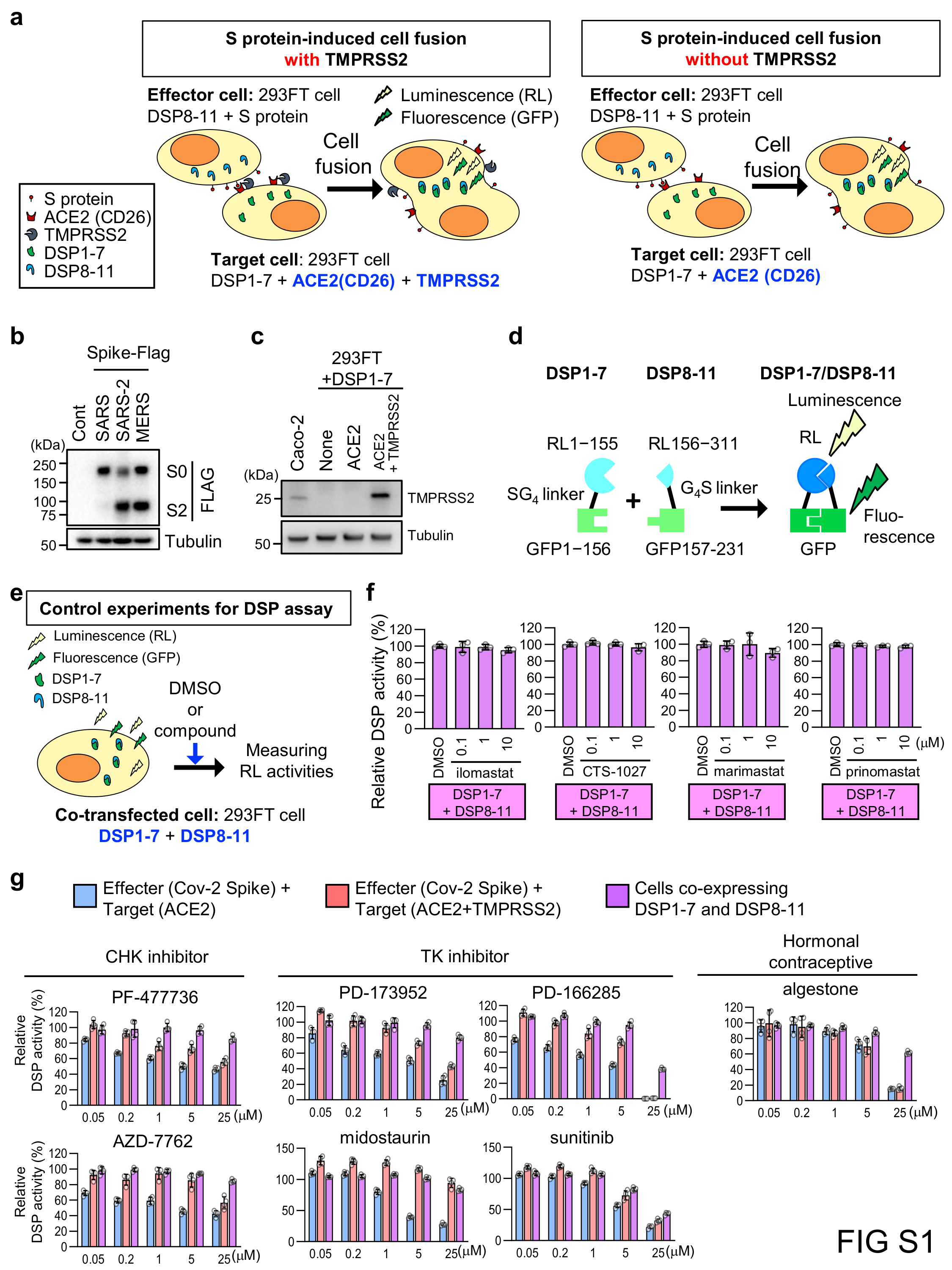

Supplement: FIG S1 [file mbio.00519-22-s0001.tif]

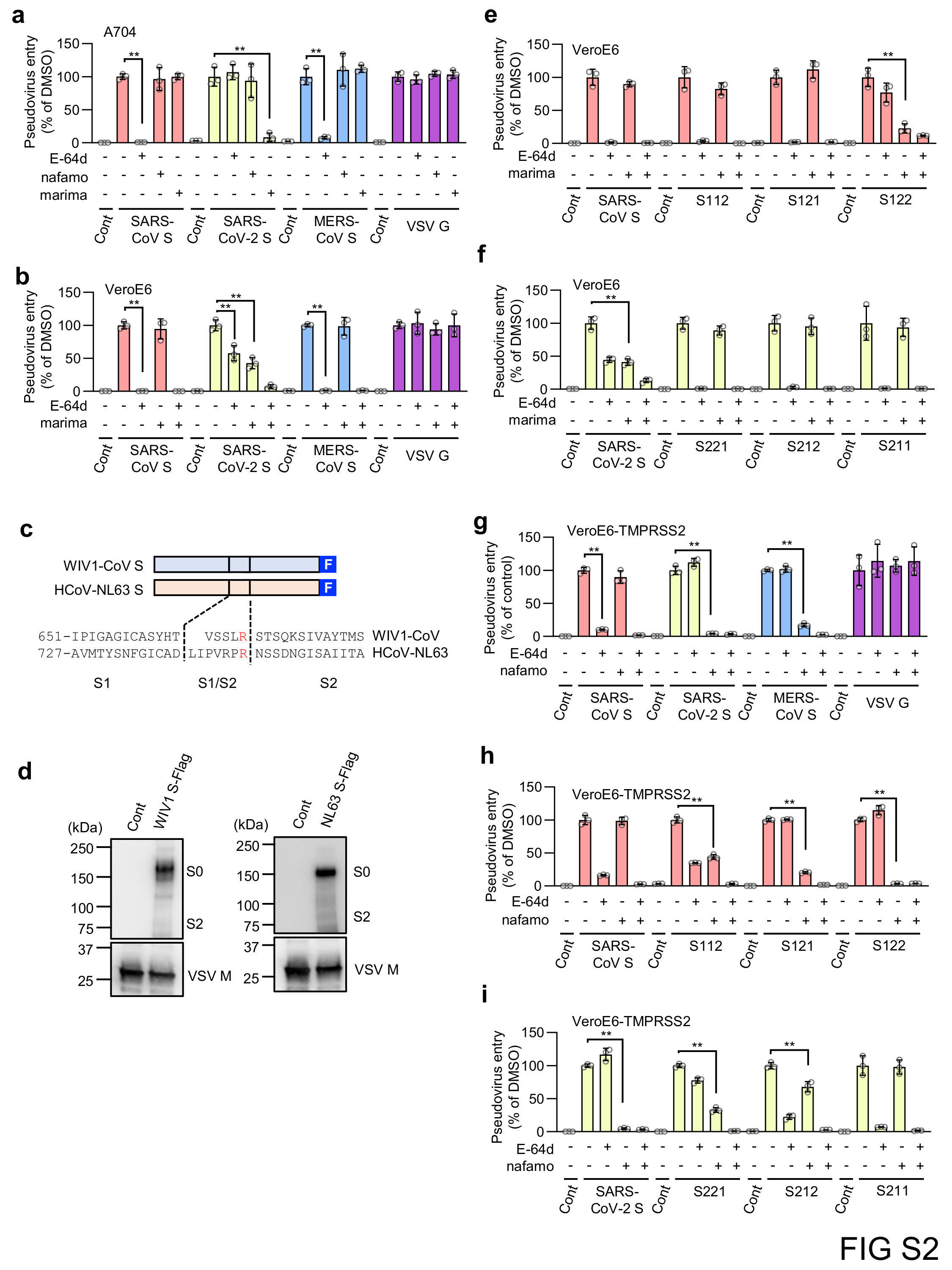

Supplement: FIG S2 [file mbio.00519-22-s0002.tif]

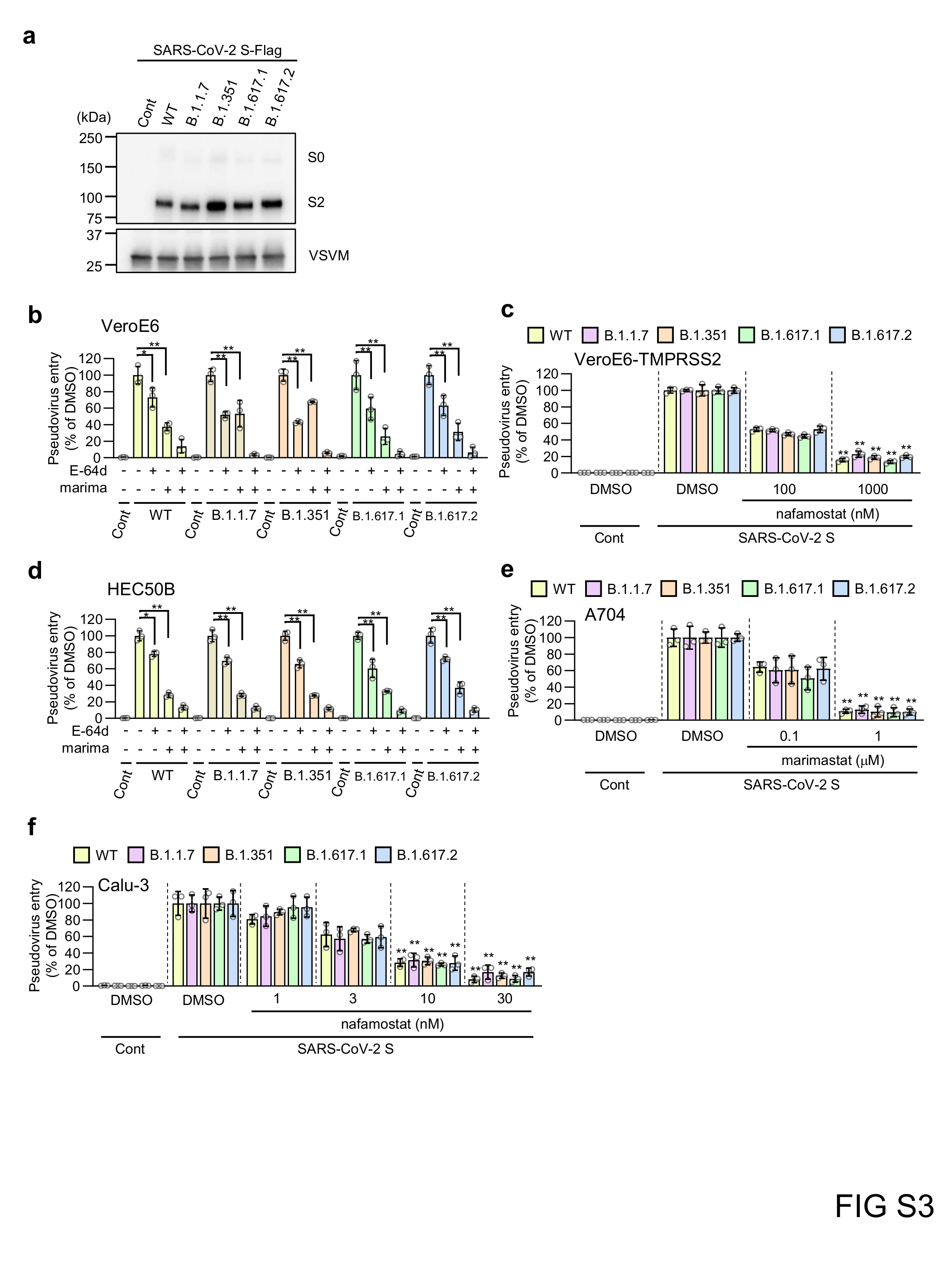

Supplement: FIG S3 [file mbio.00519-22-s0003.tif]

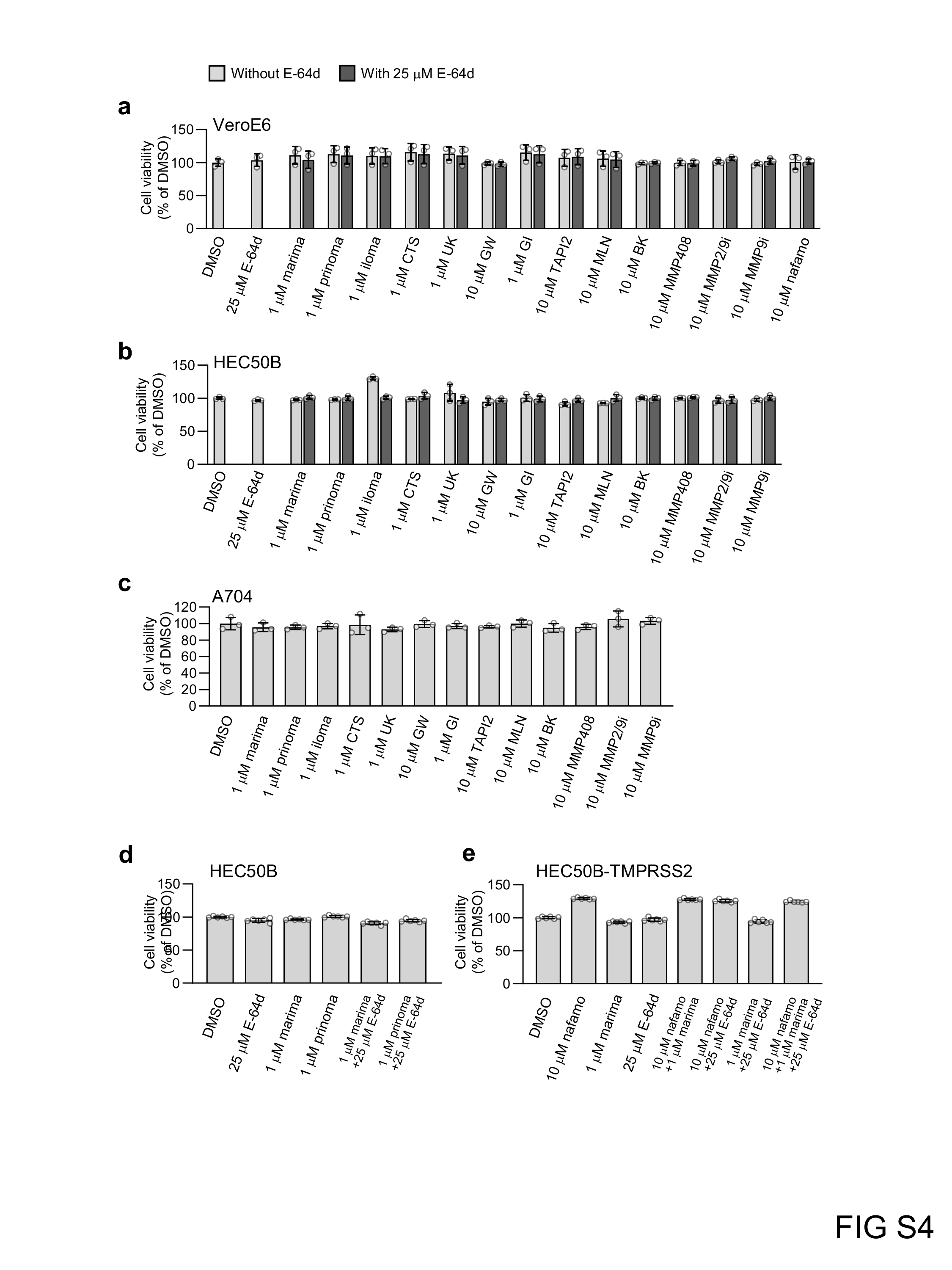

Supplement: FIG S4 [file mbio.00519-22-s0004.tif]

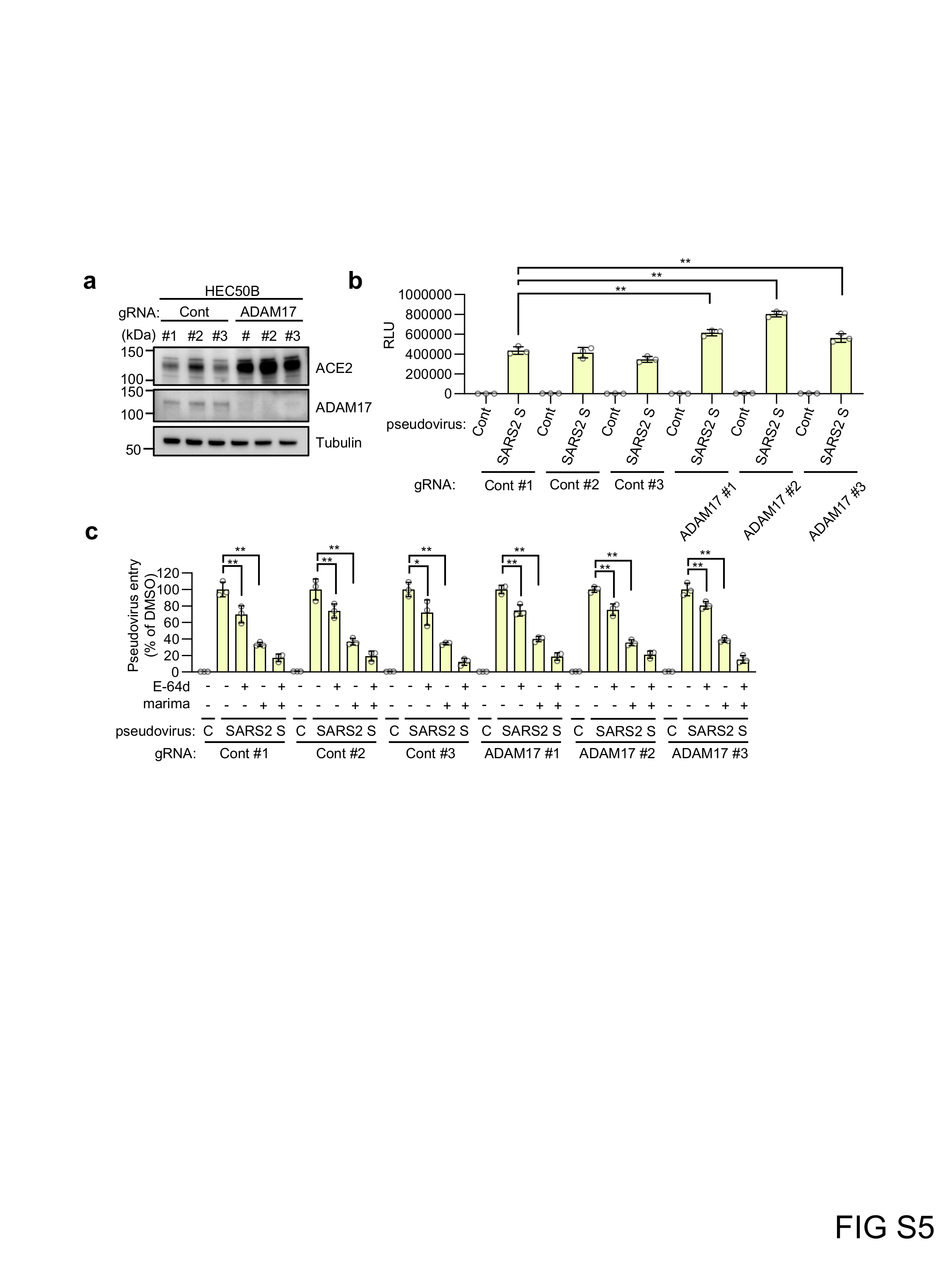

Supplement: FIG S5 [file mbio.00519-22-s0005.tif]

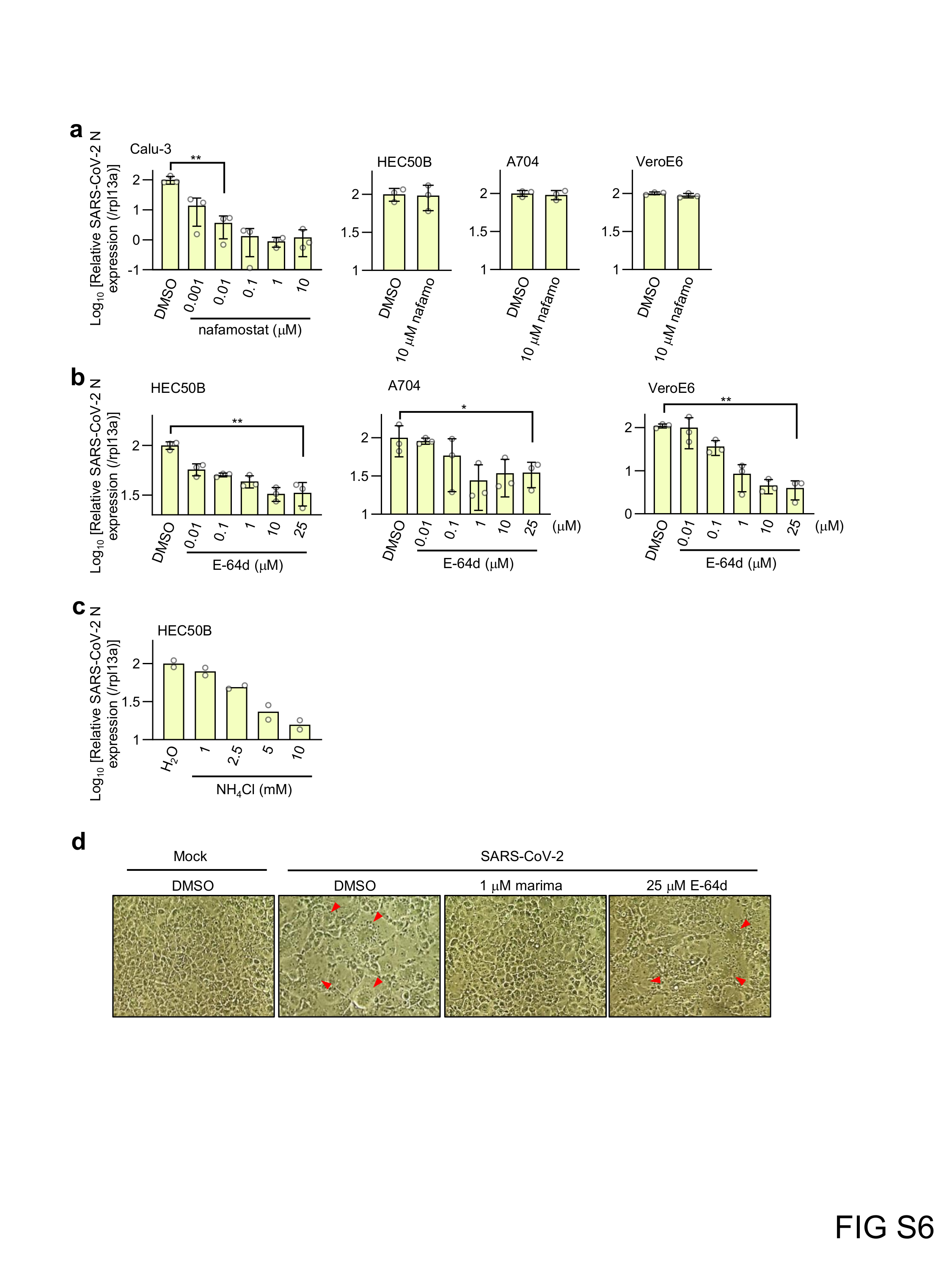

Supplement: FIG S6 [file mbio.00519-22-s0006.tif]
