# Supplementary material for: Metalloproteinase-Dependent and TMPRSS2-Independent Cell Surface Entry Pathway of SARS-CoV-2 Requires the Furin Cleavage Site and the S2 Domain of Spike Protein
Source: mBio. 2022 Jun 16;13(4):e00519-22. doi: 10.1128/mbio.00519-22 (PMC9426510; doi:10.1128/mbio.00519-22)
Supplement: TABLE S1 [file mbio.00519-22-s0007.docx]

Supplemental Table S1. Cell lines used in this study

| Cell line | Cat. Num. | Supplier | Culture condition |
| --- | --- | --- | --- |
| VeroE6 | CRL-1586 | ATCC | EMEM + 15% FBS |
| 293T | CRL-3216 | ATCC | DMEM + 10% FBS |
| A704 | HTB-45 | ATCC | EMEM + 15% FBS |
| Calu-3 | HTB-55 | ATCC | EMEM + 15% FBS |
| OVTOKO | JCRB1048 | JCRB | RPMI + 10% FBS |
| OVISE | JCRB1043 | JCRB | RPMI + 10% FBS |
| HEC50B | JCRB1145 | JCRB | EMEM + 15% FBS |
| VeroE6-TMPRSS2 | JCRB1819 | JCRB | DMEM + 10% FBS + 1 mg/ml G418 |
| OUMS-23 | JCRB1022 | JCRB | DMEM + 10% FBS |
| IGROV1 | SCC203 | Merck (Darmstadt, Germany) | DMEM + 10% FBS |
| Caco-2 | RCB0988 | RIKEN | EMEM + 15% FBS |
| 293FT | R70007 | Thermo Fisher Scientific (MA, USA) | DMEM + 10% FBS + 1 ug/mL puromycin |

ATCC, American Type Culture Collection (Rockville, MD, USA)

JCRB, Japanese Collection of Research Bioresources Cell Bank (Osaka, Japan)

RIKEN, RIKEN BioResource Research Center (Tsukuba, Japan)

EMEM, Eagle's minimum essential medium (EMEM; 055-08975, FUJIFILM Wako Pure Chemical, Osaka, Japan)

DMEM, Dulbecco’s modified Eagle’s medium (DMEM; 041-30081, FUJIFILM Wako Pure Chemical)

RPMI, Roswell Park Memorial Institute (RPMI)-1640 medium (189-02025, FUJIFILM Wako Pure Chemical)
