# Supplementary material for: Metalloproteinase-Dependent and TMPRSS2-Independent Cell Surface Entry Pathway of SARS-CoV-2 Requires the Furin Cleavage Site and the S2 Domain of Spike Protein
Source: mBio. 2022 Jun 16;13(4):e00519-22. doi: 10.1128/mbio.00519-22 (PMC9426510; doi:10.1128/mbio.00519-22)
Supplement: TABLE S2 [file mbio.00519-22-s0008.docx]

Supplemental Table S2. siRNAs and primers used in this study

| Oligonucleotide | | sequence | Supplier | |
| --- | --- | --- | --- | --- |
| siRNA | siADAM10#1 | 5'-UCA CCU UGU UCU ACC AUU CCA-3' | Thermo Fisher Scientific (MA, USA) | S1004 |
|  | siADAM10#2 | 5'-UAA CCU CUA AAA UCG UUG CAA-3' |  | S1005 |
|  | siADAM10#3 | 5'-UAC GGA UUC CGG AGA AGU CTG-3' |  | S1006 |
|  | Negative control No.1 siRNA | |  | 4390843 |
|  | Negative control No.2 siRNA | |  | 4390846 |
| Primer | SARS-CoV-2 N-Fow | 5'-AAA TTT TGG GGA CCA GGA AC-3' | | |
|  | SARS-CoV-2 N-Rev | 5'-TGG CAG CTG TGT AGG TCA AC-3' | | |
|  | Human Rpl13a-Fow | 5'-TGT TTG ACG GCA TCC CAC-3' | | |
|  | Human Rpl13a-Rev | 5'-CTG TCA CTG CCT GGT ACT TC-3' | | |
|  | African green monkey Rpl13a-Fow | 5'-CTC AAG GTT GTG CGT CTG AA-3' | | |
|  | African green monkey Rpl13a-Rev | 5'-CTG TCA CTG CCT GGT ACT TCC A-3' | | |
