# Supplementary material for: Metalloproteinase-Dependent and TMPRSS2-Independent Cell Surface Entry Pathway of SARS-CoV-2 Requires the Furin Cleavage Site and the S2 Domain of Spike Protein
Source: mBio. 2022 Jun 16;13(4):e00519-22. doi: 10.1128/mbio.00519-22 (PMC9426510; doi:10.1128/mbio.00519-22)
Supplement: TABLE S3 [file mbio.00519-22-s0009.docx]

Supplemental Table S3. Inhibitors used in this study

| Inhibitor | Supplier | |
| --- | --- | --- |
| nafamostat mesylate | Tokyo Chemical Industry (Tokyo, Japan) | N0959 |
| pepstatin A | Peptide institute (Osaka, Japan) | 4397 |
| leupeptin | Peptide institute | 4041 |
| E-64d | Peptide institute | 4321-v |
| bestatin | FUJIFILM Wako Pure Chemical (Osaka, Japan) | 027-14101 |
| furin inhibitor II | Merck (Darmstadt, Germany) | 344931 |
| MLN-4760 | Merck | 530616 |
| ilomastat | MedChemExpress (NJ, USA) | HY-15768 |
| CTS-1027 | MedChemExpress | HY-10398 |
| marimastat | MedChemExpress | HY-12169 |
| prinomastat hydrochloride | Sigma-Aldrich (MO, USA) | PZ0198 |
| GI254023X | Sigma-Aldrich | SML0789 |
| UK370106 | Tocris (MN, USA) | 2900 |
| GW280264X | Cayman (MI, USA) | 31388 |
| TAPI-2 | Cayman | 14695 |
| BK-1361 | ProbeChem (Shanghai, China) | PC-60981 |
| MMP408 | Millipore (MA, USA) | 444291 |
| MMP9 inhibitor I | Millipore | 444278 |
| MMP2/9 inhibitor I | Abcam (Cambridge, UK) | ab145190 |
| Validated Compound Library | Drug Discovery Initiative (The University of Tokyo) | |
