# Supplementary material for: Metalloproteinase-Dependent and TMPRSS2-Independent Cell Surface Entry Pathway of SARS-CoV-2 Requires the Furin Cleavage Site and the S2 Domain of Spike Protein
Source: mBio. 2022 Jun 16;13(4):e00519-22. doi: 10.1128/mbio.00519-22 (PMC9426510; doi:10.1128/mbio.00519-22)
Supplement: TABLE S4 [file mbio.00519-22-s0010.docx]

Supplemental Table S4. Antibodies used in this study

| **Antibody** | **dilution** | **Supplier** | |
| --- | --- | --- | --- |
| **For Western Blotting** | | | |
| rabbit anti-ACE2 | 1:1000 | Abcam (Cambridge, UK) | ab15348 |
| rabbit anti-TACE | 1:1000 | Cell Signaling Technology (MA, USA) | 3976S |
| rabbit anti-ADAM10 | 1:1000 | Cell Signaling Technology | 14194S |
| rabbit anti-Flag-tag | 1:1000 | MBL (MA, USA) | PM020 |
| mouse anti-tubulin | 1:1000 | Millipore (MA, USA) | CP06 |
| mouse anti-VSVM | 1:1000 | Absolute antibody (Oxford, UK) | 23H12 |
| rabbit anti-TMPRSS2 | 1:1000 | Abcam | ab109131 |
| HRP-linked donkey anti-rabbit IgG antibody | 1:1000 | GE Healthcare (Piscataway, NJ, USA) | NA934 |
| HRP-linked donkey anti-mouse IgG antibody | 1:1000 | GE Healthcare | NA931V |
| **For Immunofluorescence Staining** | | | |
| rabbit anti-SARS-CoV-2 nucleocapsid | 1:1000 | GeneTex (CA, USA) | GTX135357 |
| goat anti-rabbit-IgG-Alexa488 | 1:200 | Invitrogen (CA, USA) | A11008 |
